# Chemical composition and biological effects of kratom (*Mitragyna speciosa*): In vitro studies with implications for efficacy and drug interactions

**DOI:** 10.1038/s41598-020-76119-w

**Published:** 2020-11-05

**Authors:** D. A. Todd, J. J. Kellogg, E. D. Wallace, M. Khin, L. Flores-Bocanegra, R. S. Tanna, S. McIntosh, H. A. Raja, T. N. Graf, S. E. Hemby, M. F. Paine, N. H. Oberlies, N. B. Cech

**Affiliations:** 1 Department of Chemistry and Biochemistry, The University of North Carolina Greensboro, 435 Sullivan Bldg., 301 McIver St., Greensboro, NC 27402 USA; 2 Department of Veterinary and Biomedical Sciences, Pennsylvania State University, University Park, PA 16802 USA; 3 Department of Chemistry, The University of North Carolina Chapel Hill, Chapel Hill, NC 27599 USA; 4 Department of Pharmaceutical Sciences, Washington State University, Spokane, WA 99202 USA; 5 Department of Basic Pharmaceutical Sciences, High Point University, High Point, NC 27268 USA

**Keywords:** Drug discovery, Plant sciences, Health care, Chemistry

## Abstract

The safety and efficacy of kratom *(Mitragyna speciosa*) for treatment of pain is highly controversial. Kratom produces more than 40 structurally related alkaloids, but most studies have focused on just two of these, mitragynine and 7-hydroxymitragynine. Here, we profiled 53 commercial kratom products using untargeted LC–MS metabolomics, revealing two distinct chemotypes that contain different levels of the alkaloid speciofoline. Both chemotypes were confirmed with DNA barcoding to be *M. speciosa.* To evaluate the biological relevance of variable speciofoline levels in kratom, we compared the opioid receptor binding activity of speciofoline, mitragynine, and 7-hydroxymitragynine. Mitragynine and 7-hydroxymitragynine function as partial agonists of the human µ-opioid receptor, while speciofoline does not exhibit measurable binding affinity at the µ-, δ- or ƙ-opioid receptors. Importantly, mitragynine and 7-hydroxymitragynine demonstrate functional selectivity for G-protein signaling, with no measurable recruitment of β-arrestin. Overall, the study demonstrates the unique binding and functional profiles of the kratom alkaloids, suggesting potential utility for managing pain, but further studies are needed to follow up on these in vitro findings. All three kratom alkaloids tested inhibited select cytochrome P450 enzymes, suggesting a potential risk for adverse interactions when kratom is co-consumed with drugs metabolized by these enzymes.

## Introduction

According to the US Department of Health and Human Services, opioids were responsible for more than 42,000 deaths in the US in 2016, the highest in recorded history^1^. More than 40% of these deaths involved a prescription opioid. Some individuals who suffer from chronic pain are turning to other options, one of which is the plant known as kratom [*Mitragyna speciosa* (Korth.) Havil. (Rubiaceae)], a tropical tree native to peninsular Thailand, Myanmar, Malaysia and other countries in Southeast Asia^1,2^. Kratom has skyrocketed in popularity in western countries in the past decade; current estimates are that as many as 5 million individuals in the US use kratom on a regular basis^3^. There has been considerable controversy over the safety and efficacy of kratom use by US consumers. Citing safety concerns (one study reports that kratom has been implicated as at least partially involved in 91 deaths)^4^, the United States Department of Agriculture (USDA) has made it their practice to confiscate shipments of kratom into the US. The US Drug Enforcement Administration (DEA) threatened to assign kratom as a schedule 1 controlled substance, which would make possession of kratom illegal. The DEA then suspended that decision in response to a backlash from some US consumers^5^, who claim that it is a safer alternative to opioids for treatment of pain and/or opioid addiction^6^. The effectiveness of kratom for these purposes continues to be a hotly debated and politically charged topic, and one that requires rigorous scientific investigation.

Widespread kratom use is a relatively new phenomenon in the US^6^. However, the medicinal use of kratom in southeast Asia has a long history. Kratom was first documented in the scientific literature in 1836^7^, in a paper that described use of kratom leaves by the Malays as a substitute for opium. The isolation of mitragynine was reported in 1921 by Ellen Field^8^, who ended the introduction of her paper with the following statements, “*According to Redley………..Mitragyne [sic] speciosa is used in Perak against the opium habit, whilst, according to Dr. P.P. Laidlaw, mitragynine is a local anaesthetic [sic]…*” Thus, controversy over the use and effectiveness of kratom was documented nearly 100 years ago. It is a controversy that continues today.

**Figure 1:**
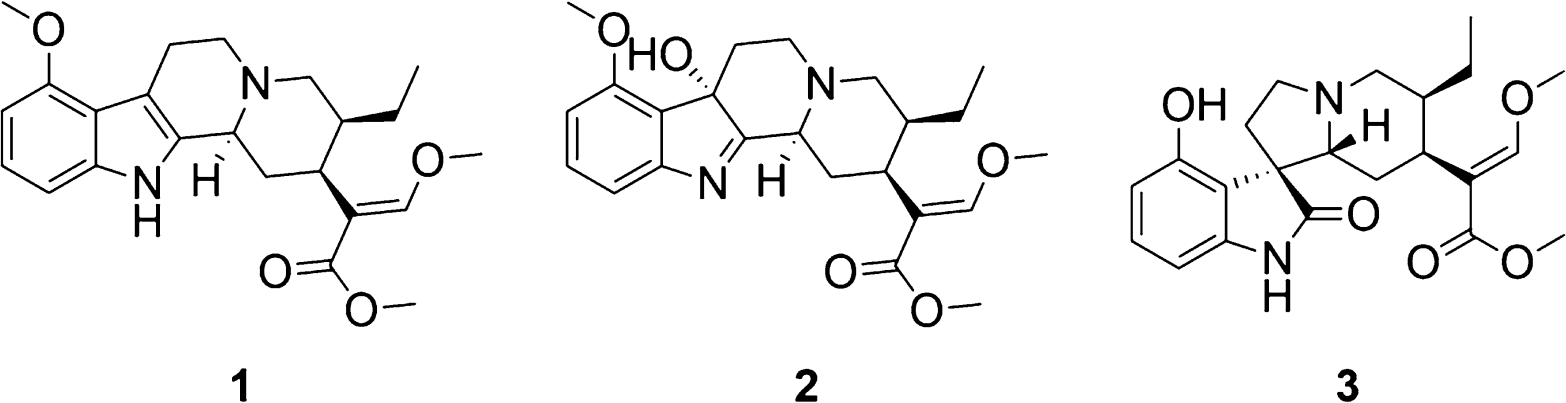
Structures of select alkaloids present in kratom (*Mitragyna speciosa*), mitragynine (**1**), 7-hydroxymitragynine (**2**) and speciofoline (**3**).

Consistent with the claim that kratom can be effective in the treatment of pain, extracts from this plant demonstrated opioid-receptor mediated analgesic effects in mouse model studies^9^. This activity has generally been attributed to alkaloids that the plant produces, of which mitragynine (**1**) and 7-hydroxymitragynine (**2**) (Fig. 1) have been the focus of multiple pharmacological investigations^10–13^. Mitragynine and 7-hydroxymitragynine both bind to the human µ-opioid and ƙ-opioid receptors (hMOR, hKOR) with nanomolar affinity, and function as partial agonists at the µ-opioid receptor and weak antagonists at ƙ-opioid and δ-opioid receptors^14,15^. 7-Hydroxymitragynine exhibits approximately fivefold greater affinity at the μ-opioid receptor compared to mitragynine. Upon receptor activation, mitragynine and 7-hydroxymitragynine exhibit functional selectivity for G-protein signaling, with no measurable recruitment of β-arrestin^14^. In antinociception assays, 7-hydroxymitragynine exhibits 40-fold greater potency than mitragynine and tenfold greater potency than morphine^16,17^, whereas mitragynine is less potent than morphine in antinociceptive assays^18^. Combined administration of mitragynine with morphine increases antinociception compared with morphine alone and prevents the development of morphine tolerance^19^. In contrast to mitragynine, repeated administration of 7-hydroxymitragynine produces antinociceptive tolerance as well as cross-tolerance to morphine’s antinociceptive action and induces physical dependence^13^. Furthermore, mitragynine does not exhibit abuse liability and decreases the reinforcing effects of morphine whereas 7-hyroxymitragynine demonstrates abuse liability and increased morphine self-administration in rats^10^. Interestingly, a recent study by Kruegel et al.^12^ shows that mitragynine can be converted to 7-hydroxymitragynine both in vitro and in a mouse model, therefore, some of the in vitro activity attributed to mitragynine may in fact be due to the action of it metabolite 7-hydroxymitragynine.

Collectively, the scientific data on mitragynine and 7-hydroxymitragynine suggest that kratom tea might indeed be an effective alternative to opioids against pain. However, kratom products are sold to consumers under a variety of trade names with no data regarding the chemical composition of plant material being consumed. Kratom has been documented to produce variable levels of its more than 40 alkaloids, depending on genetic variation and differences in growth and processing conditions^6,20–22^. What are the potential health implications of the variability in chemical content of kratom preparations? As of yet, this question has been difficult to answer because mechanistic studies of kratom alkaloids have focused largely on isolated alkaloids without consideration of how these alkaloids are represented in kratom materials used medicinally. Herein, we sought to connect the dots between investigations of pure alkaloids and their relevance to medicinally used kratom. Towards this goal, we employed untargeted metabolomics to determine which alkaloids vary in content across commercial kratom products. We then isolated these relevant alkaloids and compared their biological effects in vitro, including activity at the µ-opioid receptors and inhibitory effects on CYP isoforms involved in the metabolism of opioids and other drugs. Our ultimate objective with this study was to capture the variability of commercial kratom products being employed by US consumers and to evaluate (based on vitro studies) the potential implications of this variability in terms of safety and efficacy.

## Results

### Commercial kratom products can be divided into two chemotypes with differing alkaloid profiles

**Figure 2:**
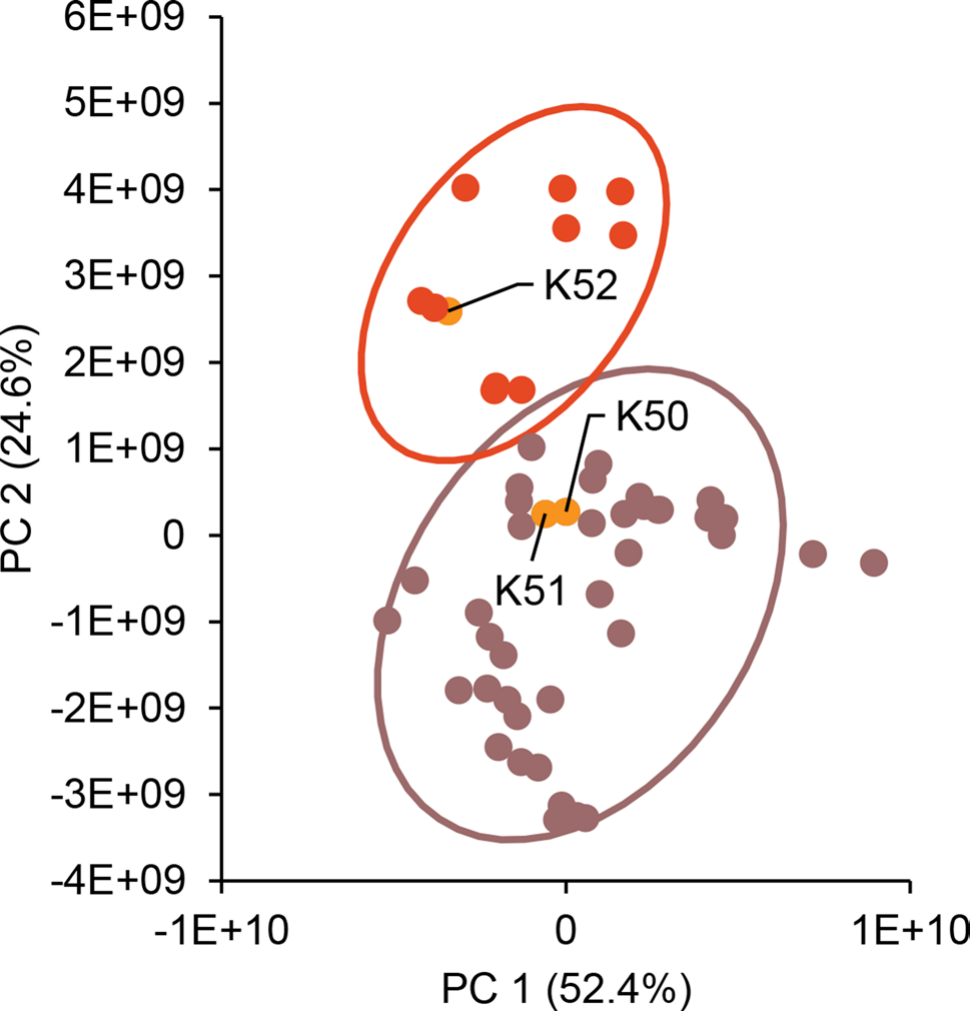
Principal component analysis (PCA) scores plot generated based on LC–MS metabolomics traces of kratom (*Mitragyna speciosa*) samples in Table 1. Each data point represents the average peak areas of triplicate extractions prepared for each sample. Samples selected for in-depth analysis herein (K50–K52) are highlighted in orange. Two groupings were evident from the initial analysis (indicated here with separate Hotelling’s T^2^ 95% confidence intervals). The underlying cause for these apparent differences in the data was further explored with loadings plot and volcano plot data (Fig. 3).

**Figure 3:**
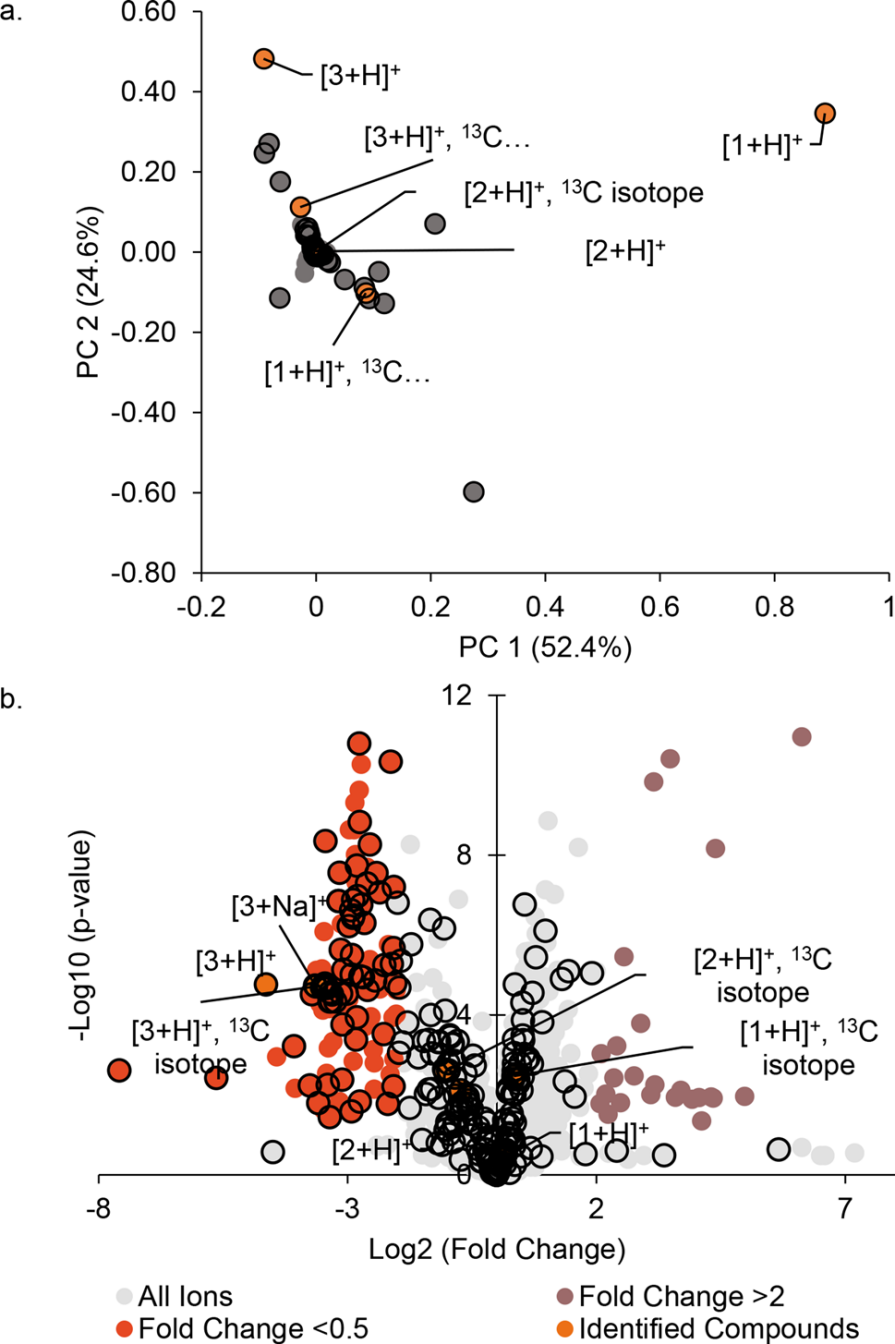
Plots of significant features based upon the PCA analysis. (**A**) Loadings plot, where the spatial arrangement of the features (unique *m/z*-RT pairings) corresponds to the distribution of the samples (i.e., scores plot, Fig. 2). Speciofoline (**3**) appears to be a feature that is responsible for discriminating between the two sample groupings along the y-axis (PC2). (**B**) Volcano plot, highlighting significant features of the metabolites based on statistical testing (p value < 0.05, fold change > 2 or < 0.5) between the two groupings, with the further a feature’s position away from the origin (0, 0), the more significant the feature is. The speciofoline-rich group (red circles) yielded 120 significant features, while 19 features were significantly distinct in the non-speciofoline-rich grouping of commercial samples (purple circles). Speciofoline (**3**) was found to be a metabolite that differed significantly across sample groups, while mitragynine (**1**) and 7-hydroxymitragynine (**2**) were not significantly different between the two groups (orange circles). Other features predicted to be alkaloids based on mass defect (open circles). Labels on plot refer to the compound number and the type of ion observed (i.e.[3 + H]^+^ refers to the protonated molecule of speciofoline).

We conducted untargeted metabolomics analysis of more than 50 commercial available kratom products (Table S1) to obtain a global comparison of chemical composition. A principal component analysis (PCA) plot of the resulting data (Fig. 2) reveals what appear to be two different groupings of the data. It was of interest to determine which chemical constituent(s) were responsible for these differences. To do so, we examined loadings and volcano plots (Fig. 3) and determined that the peak area of a constituent with *m/z* of 401.2064 and retention time of 2.70 varied across the two groups. This constituent was isolated and identified as the known kratom oxindole alkaloid speciofoline (**3**, Fig. 1) ([M + H]^+^  = 401.2071, Δ = 1.7 ppm) based on comparison of NMR, ECD, and mass spectrometry data with literature values^23–26^ (Table S2, Figure S1 and S2). Several other ions were also observed to vary in content across the samples (Fig. 3), and based on their similar mass defect to indole alkaloids^27^, are likely other more minor alkaloids of kratom. Structures of these other putative alkaloids were not determined in this study.

**Figure 4:**
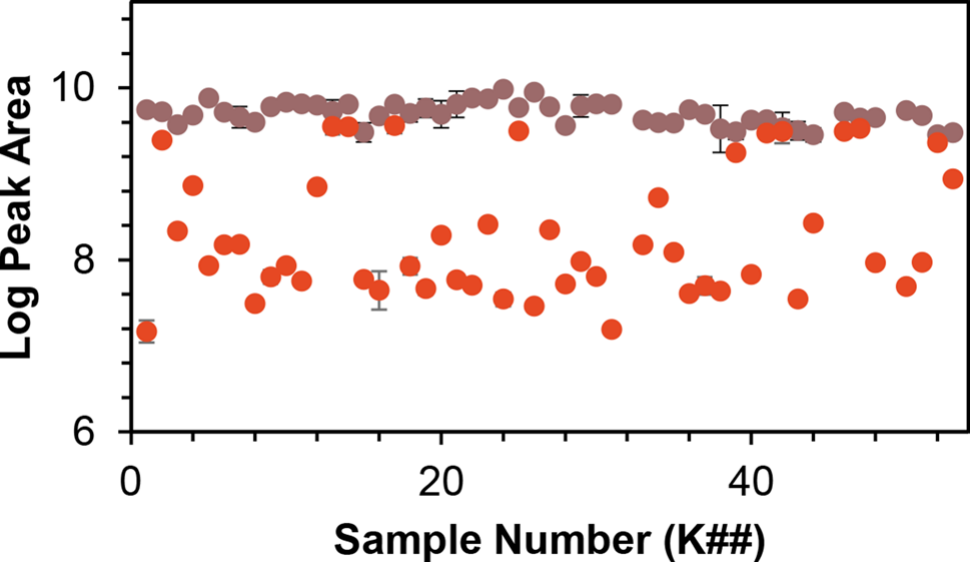
Comparison of mitragyine and speciofoline levels across samples of *M. speciosa*. The x-axis represents arbitrary codes assigned to the samples, as described in Table S1, and the y-axis represents the log of the peak area for the relevant selected ion trace in LC–MS analysis.

Based on the chemical data (Fig. 3), it appears that the commercial kratom products studied herein can be grouped into separate “high-speciofoline” and “low-speciofoline” chemotypes. To demonstrate this further, a comparison is shown of chromatographic peak area across all samples for the ions corresponding to mitragynine and speciofoline (Fig. 4). The mitragynine content in the commercial samples varied by fourfold, while the speciofoline content varied by more than 90-fold, with a minority of samples appearing as “high-speciofoline” outliers (Fig. 4).

To further explore the phenomenon of variable speciofoline levels in kratom samples, we utilized the isolated speciofoline to conduct quantitative analysis for representative high and low-speciofoline materials (Table 1). A limitation of working with commercial kratom samples is that morphological information that could be used to identify the plants is lost when they are powdered. To address this limitation, we obtained a young, living plant from a supplier in Ohio, which appeared based on morphological characteristics to be authentic *M. speciosa*. This sample was coded as K55 and was included in the quantitative analysis.

**Table 1:** Alkaloid quantity in Kratom products.

Sample code	Mitragynine (mg/g)^b^	7-Hydroxymitragynine (mg/g)^b^	Speciofoline (mg/g)^b^
K50	11.37 ± 0.83	0.053 ± 0.014	< LOQ^c^
K51	11.45 ± 0.74	0.056 ± 0.019	< LOQ^c^
K52	8.13 ± 0.95	0.0349 ± 0.0030	4.13 ± 0.45
K55	0.75 ± 0.14	< LOQ^d^	< LOQ^c^

Samples K50, K51 and K52 are commercial kratom products that were purchased for this study. Sample K55 is a young cultivated kratom plant. Quantities were calculated by preparing methanolic extracts in triplicate and analyzing by LC–MS.

^a^Quantity is denoted as mg of compound per g of dried kratom plant material.

^b^Uncertainty calculated using standard deviation of triplicate extractions, each analyzed separately.

^c^Limit of quantitation (LOQ) for speciofoline is 4.9 ng/mL (Table S3) and this sample contained speciofoline below that level.

^d^LOQ for 7-hydroxymitragynine is 0.49 ng/mL (Table S3) and this sample contained 7-hydroxymitragynine below that level.

As expected, the quantities of alkaloids differ in the two commercial kratom products subjected to quantitative analysis (K51 and K52). Sample K51 contained mitragynine at a level of 11.45 ± 0.74 mg/g (1.145 ± 0.074%), and 7-hydroxymitragynine at a level of 0.056 ± 0.019 (0.0056 ± 0.002%). These values are consistent with prior literature^28^, which found that mitragynine was detected in commercial kratom products at a concentration range between 1 and 6% of leaf content and that 7-hydroxymitragynine levels ranged from 0.01 to 0.04% of leaf content. In contrast, sample K52 contains lower levels of mitragynine (8.13 ± 0.95 mg/g) but, as expected based on the data in Fig. 4, has a much higher content of speciofoline (Table 1) than sample K51. Interestingly, the kratom plant used for morphological identification (K55) showed much lower abundance of mitragynine and 7-hydroxymitragynine than either of the commercial kratom samples (K51 and K52). The reason for this difference cannot be ascertained with the experimental design employed for this study, but may be related to differences in plant age and/or growing conditions for K55 as compared to the commercial kratom products. Because of the markedly low alkaloid levels (Table 1), the K55 kratom sample was employed only for genetic studies and was not further evaluated for pharmacological activity.

### Genetic differences do not appear to explain the observed chemotypes in commercial kratom samples

There are 10 related species within the *Mitragyna* genus^6^, including *Mitragyna speciosa* (Korth.) Havil., *Mitragyna diversifolia* (Wall. ex G. Don) Havil., *Mitragyna hirsuta* Havil., and *Mitragyna rotundifolia* (Roxb.) Kuntze. While these species can be distinguished by morphological characteristics in raw plant material, contamination or misidentification could occur during collection and could not be distinguished in powdered material such as that used in this study. The dramatic differences in alkaloid quantity in various kratom samples demonstrated in Table 1 and Fig. 4 led us to ask whether the samples reported to be *Mitragyna speciosa* might be different species or strains of *Mitragyna.* To address this question, we extracted DNA from three kratom samples with varying speciofoline content (K51, K52 and K55 in Table 1), and used the resulting data for DNA barcoding via BLAST search of *rbcL* + *matK* (core plant barcoding markers) and performed maximum likelihood and Bayesian phylogenetic analysis, of the combined *matK* + *trnH-psbA* and the separate nrDNA ITS regions. These regions were selected because they are designated as the official DNA barcodes for the plant kingdom^29–37^.

Molecular identification of plant samples using a BLAST search of core plant loci (*rbcL* + *matK*) using the BOLDSYSTEMS database (version 4) showed that samples K51, K52 and K55 had ≥ 99% sequence similarity with *M. speciosa* (Figures S4–S6). Based on uncorrected p distances calculated for *trnH-psbA*, there was ≥ 99–100% sequence similarity with sequences of published *M. speciosa.* This means that samples K51, K52 and K55 were more similar to sequences of *M. speciosa* and more dissimilar to sequences of *M. diversifolia, M. hirsuta, M. rotundifolia*, and *M. parvifolia* (Table S5a and b). Maximum likelihood analysis of the partial *matK* + *trnH-psbA* fragments placed all three samples in a strongly supported clade (100% RAxML bootstrap support, 78% PhyML bootstrap and ≥ 95% Bayesian posterior probability support) with the published sequences for *M. speciosa* (Figure S7). Notably, this analysis included a partial *matK* sequence from a recently sequenced genome of *M. speciosa* (BioProject: PRJNA325670). Finally, phylogenetic analysis using the nrDNA ITS region also placed K51 and K52 in a strongly supported clade (≥ 84% PhyML bootstrap and ≥ 95% Bayesian posterior probability support) with *M. speciosa* sequences (Figure S8). However, the ITS analysis showed that K55 does not group with *M. speciosa*, but rather formed a cluster with other *Mitragyna* spp., albeit without any significant bootstrap support and/or Bayesian posterior probability support (Figure S8).

The results of DNA barcoding and phylogenetic analyses classify all three kratom products as *Mitragyna speciosa*, based on comparison with the published sequences for this species, except for the ITS analysis, which showed a conflicting outcome for sample K55. Thus, it appears that the observed differences in speciofoline content among the various kratom samples is not due to misidentification of species or substitution of a different *Mitragyna* species for *M. speciosa*. Notably, a number of other factors including plant age, growing conditions, and processing methods could all contribute to the observed differences in alkaloid level in the various kratom samples. Exploring these differences would require a different experimental design and could be the subject of future studies. For the purpose of the current investigation, the important finding is that dramatic differences in alkaloid content can be observed among different samples of what appear to be authentic (based on molecular data) *M. speciosa*. Such differences in alkaloid profile could have implications for biological activity, a possibility that we explore in the next sections.

### Differences in alkaloid profile in kratom plant material is expected to have implications for opioid receptor-mediated activity

**Figure 5:**
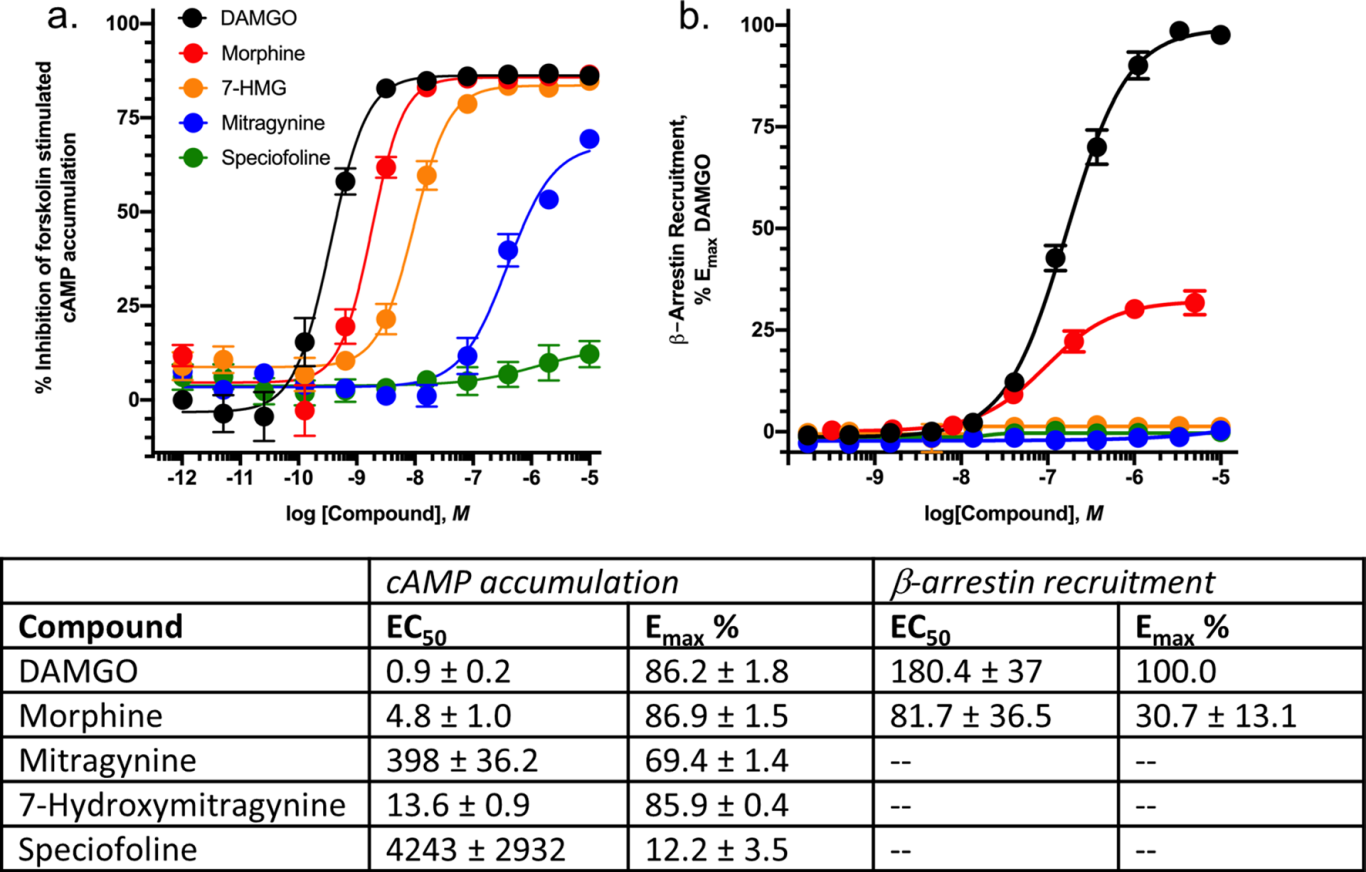
Kratom alkaloid mediated inhibition of forskolin-stimulated cAMP accumulation and β-arrestin recruitment. (**A**) CHO-K1 cells stably expressing hMOR were incubated with the indicated concentrations of compounds as described in Methods. Data are expressed as the percent of forskolin-stimulated cAMP accumulation and represent mean ± SEM of 3–4 experiments. (**B**) β-arrestin-2 recruitment. CHO-K1 cells stably expressing the µ-opioid receptor and β-galactosidase linked β-arrestin-2 were incubated with the indicated concentrations of compounds as described in Methods. Data are expressed as the percent of maximal DAMGO mediated response and represent mean ± SEM of 3–4 experiments. E_max_ and EC_50_ values for cAMP accumulation and β-arrestin2 recruitment are listed in the table.

The alkaloid speciofoline is an oxindole, while the more commonly studied alkaloids mitragynine and 7-hydroxymitragynine are indoles (Scheme 1). Thus, we hypothesized that there might be biological implications to the observed variability in speciofoline levels across kratom extracts (Table 1). To explore this hypothesis, we tested mitragynine (**1**), 7-hydroxymitragynine (**2**) and speciofoline (**3**) for µ-opioid receptor inhibition of forskolin-stimulated cyclic-AMP (cAMP) accumulation and for their ability to recruit β-arrestin-2 in CHO-K1 cells stably expressing the µ-opioid receptor. Results indicate that DAMGO and morphine fully inhibit cAMP accumulation, mitragynine and 7-hydroxymitragynine partially inhibit cAMP accumulation, whereas speciofoline has no effect in this assay (Fig. 5A). Furthermore, DAMGO and morphine effectively recruit β-arrestin-2 in CHO cells (Fig. 5B), while mitragynine, 7-hydroxymitragynine, and speciofoline fail to recruit β-arrestin-2 at concentrations as high as 10 mM.

To further characterize the pharmacology of the kratom alkaloids, competitive radioligand binding assays were conducted with HEK293 cells stably expressing µ-, ƙ- and δ-opioid receptors to assess the affinity of the alkaloids at these receptors (Table 2). Mitragynine exhibited moderate affinity at µ- and ƙ-opioid receptors whereas 7-hydroxymitragynine showed strong affinity at the µ- opioid receptor (14X greater than mitragynine) and moderate affinity at the ƙ- and δ-opioid receptors (4X and 70X greater than mitragynine). In contrast, speciofoline did not exhibit appreciable affinity for any of the opioid receptors. Results for mitragynine and 7-hydroxymitragynine are consistent with previous literature reports^14,15^; however, this is the first published assessment of receptor binding of speciofoline at opioid receptors.

**Table 2:** Opioid receptor affinities for kratom alkaloids.

Compound	Receptor binding affinity (Ki ± SEM, nM)		
	µ-opioid	ƙ-opioid	δ-opioid
Mitragynine	238 ± 28	482 ± 29	> *10,000*
7-Hydroxymitragynine	16 ± 1	113 ± 37	137 ± 21
Speciofoline	> *10,000*	> *10,000*	> *10,000*
Morphine	1.50 ± 0.04	48.9 ± 5.0	> *10,000*

Competitive binding studies were performed against ^3^H-DAMGO, ^3^H-U69-593 and ^3^H-deltorphin for µ-, ƙ-,and δ-opioid receptors respectively. Results are presented as nM ± SEM from a minimum of three replicates.

The differential binding affinity of mitragynine and 7-hydroxymitragynine at opioid receptors (Table 2) is consistent with previous literature^14,15^. Also consistent with literature reports^14,15^ both mitragynine and 7-hydroxymitragynine function as partial agonists at the µ-opioid receptors and are functionally selective for G protein versus β-arrestin-2 signaling (Fig. 5). These effects are quite different from the binding and activity of typical opioids such as morphine (Fig. 5). The failure of mitragynine or 7-hydroxymitragynine to recruit β-arrestin-2 may be an important factor separating the analgesic effect from the adverse effects of typical opiates such as abuse liability, respiratory depression and constipation. However, 7-hydroxymitgraynine, but not mitragynine, exhibits abuse liability in rodent models of human drug-taking^18^, suggesting that β-arrestin-2 signaling may not be a significant contributing factor to the addictive properties of opiates. In contrast, speciofoline does not bind at the opioid receptors and does not exhibit functional activity via the µ-opioid receptor. Future studies are warranted to characterize speciofoline and determine if the ligand may induce antinociception or other activities through non-opioid receptor systems, and to further characterize the pharmacological effects of kratom extracts and their isolated alkaloids in vivo.

### High-speciofoline and low-speciofoline kratom extracts have similar inhibitory effects on three major CYPs in vitro

The cytochromes P450 (CYPs) constitute a superfamily of oxidative enzymes that mediate the metabolism of approximately 50% of the top 200 most prescribed drugs^38^. Inhibition of these enzymes by xenobiotics, including drugs and botanical products (precipitants), can lead to increased systemic exposure to the object drug and potential adverse or toxic effects. Kratom extracts^39^ and mitragynine^11,40^ have been tested as inhibitors of several CYPs, including CYP1A2, CYP2C9, CYP2C19, CYP2D6, and CYP3A4. Tested extracts and mitragynine were generally stronger inhibitors of CYP2D6 activity relative to the other isoforms^39,40^.

**Figure 6:**
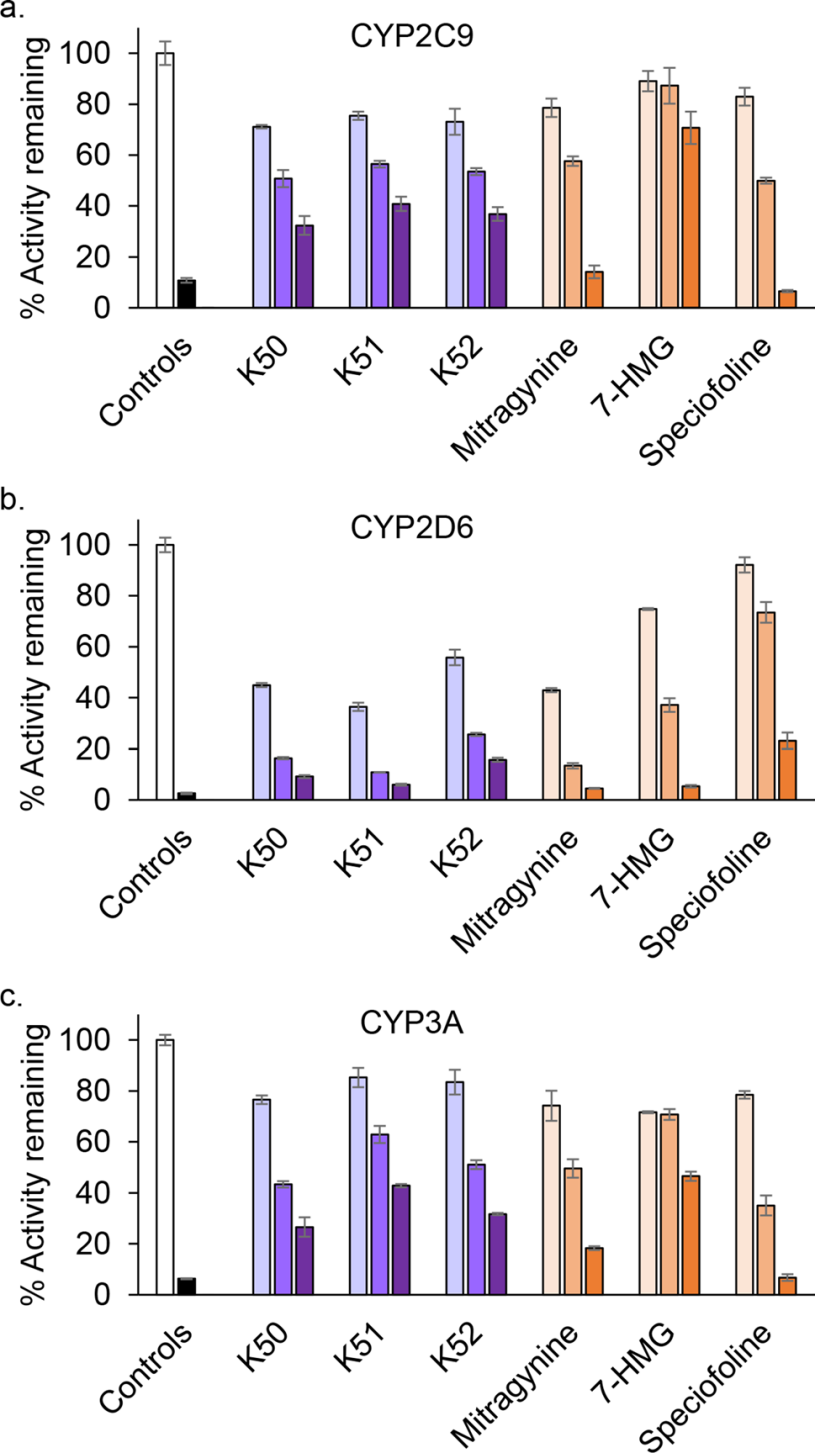
Kratom extracts (K50, K51, and K52), mitragynine, 7-hydroxymitragynine (7-HMG), and speciofoline showed concentration-dependent inhibition of cytochrome P450 (CYP) enzymes in human liver microsomes. Diclofenac 4′-hydroxylation (**A**), dextromethorphan *O*-demethylation (**B**), and midazolam 1′-hydroxylation (**C**) were used as measures of CYP2C9, CYP2D6, and CYP3A activity, respectively. Extracts were tested at 2, 10, and 20 µg/mL; alkaloids were tested at 1, 10, and 100 µM. Positive control inhibitors included sulfaphenazole (1 µM), quinidine (2 µM), and ketoconazole (0.1 µM) for CYP2C9, CYP2D6, and CYP3A activity, respectively. Control activities averaged 220, 70, and 1270 pmol/min/mg, respectively. Bars and error bars denote means and standard deviations, respectively, of triplicate incubations.

Mitragynine has been reported to inhibit several cytochrome P450s (CYPs)^11,39–41^, which could lead to increased systemic exposure to co-consumed drugs, including opioids, and potential adverse effects. We evaluated whether the observed variability in alkaloid profile for kratom extracts results in altered inhibitory effects on three major CYPs, specifically CYP2C9 (Fig. 6A), CYP2D6 (Fig. 6B), and CYP3A (Fig. 6C). All three kratom extracts tested showed concentration-dependent inhibition of each CYP, with stronger effects on CYP2D6 compared to CYP2C9 and CYP3A. Effects on each CYP were similar between the high speciofoline (K52) and low speciofoline (K50 and K51) kratom samples (Fig. 6). Mitragynine and speciofoline showed similar inhibitory effects towards CYP2C9 and CYP3A activity, whereas mitragynine showed stronger inhibition than speciofoline against CYP2D6 activity. Relative to mitragynine and speciofoline, 7-hydroxymitragynine was a weak inhibitor of CYP2C9 and CYP3A activity and a moderate inhibitor of CYP2D6 activity (i.e., inhibitory effects via mitragynine > 7-hydroxymitragynine > speciofoline).The concentration of mitragynine in the kratom extracts tested at 20 µg/mL was ~ 1 µM. The stronger inhibitory effects of the extracts compared to an equimolar concentration of isolated mitragynine indicated additional CYP inhibitors are present in the extracts. If the concentration of mitragynine in the liver upon kratom consumption nears the IC_50_ values estimated from the screening assay (~ 1 µM for CYP2D6; 10–100 µM for CYP2C9 and CYP3A), a CYP-mediated kratom-drug interaction is possible. In addition, because CYP3A is also expressed in the intestine, mitragynine concentrations in the intestine could well exceed 10 µM, increasing the possibility of CYP3A-mediated kratom-drug interactions. A comprehensive clinical pharmacokinetic study with a well-characterized kratom product is needed to determine the systemic exposure to mitragynine and other kratom alkaloids, as well as additional mechanistic in vitro studies, to ascertain the likelihood of pharmacokinetic kratom-drug interactions.

## Discussion

Collectively, our chemical and in vitro studies support previous studies suggesting that the purported efficacy of kratom against pain is mediated at least in part by kratom alkaloids that function as biased ligands of the µ-opioid receptors. These findings further highlight the value of pursuing mitragynine and related alkaloids as an alternative strategy to treat pain, as, consistent with previous literature, our findings suggest that they have less abuse liability than classic opiates such as morphine. Future in vivo studies are necessary to further explore this possibility.

Our results also demonstrate that the identity and abundance of major alkaloids is highly variable in genetically similar kratom plant material, with some kratom products being dominated by the indole alkaloid mitragynine and others containing high levels of both mitragynine and the oxindole alkaloid speciofoline. On the basis of our studies, it is reasonable to predict that individuals who self-administer kratom tea to treat pain, addiction, or depression might achieve very different results depending on the alkaloid profile of the product that they use. Such differences in activity would be difficult to predict for consumers, given that the variability in alkaloid content is in no way reflected in the product labeling. Additional studies are warranted to understand the underlying causes for variability in alkaloid content in kratom plant material, and to evaluate the therapeutic implications of this variability.

Consistent with previous literature, our results raise concern about potential drug interactions that may occur when kratom is consumed concurrently with opiates or other drugs metabolized by the cytochrome P450s. Following up on these findings, future clinical studies to evaluate the safety and efficacy of kratom and its alkaloids would be of great benefit to the public that may employ this botanical for the treatment of pain, currently or in the future. In such studies, it would be useful to consider the potential differences in activity that would be observed by complex *M. speciosa* preparations as compared to their isolated alkaloids, and to account for potential differences in alkaloid profile in different kratom materials. Chemical profiling of samples prior to evaluating their biological activity is clearly crucial, and it is important that the biological studies be informed by the results of the chemical analyses. This study demonstrates how untargeted mass spectrometry metabolomics followed by targeted mass spectrometric quantitation can be effectively employed to generate chemical data valuable to inform biological evaluation. As such, our approach can serve as a model for the design of future studies.

## Materials/methods

### General

DAMGO, quinidine, ketoconazole, morphine sulfate, and alprazolam were purchased from MilliporeSigma (St Louis, MO). Mitragynine, 7-hydroxymitragynine, midazolam, 1′-hydroxymidazolam, sulfaphenazole, and NADPH were obtained from Cayman Chemical Company (Ann Arbor, MI). Diclofenac, 4′-hydroxydiclofenac, dextromethorphan, and dextrorphan were obtained from Toronto Research Chemicals, Inc. (Toronto, Canada). Potassium phosphate buffer salts were obtained from Fisher Scientific (Fair Lawn, NJ). Human liver microsomes were obtained from XenoTech, LLC (H0604, mixed gender, pool from 15 donors, lot no. 1010191; Kansas City, KS). All other chemicals and reagents used were analytical grade.

### Acquisition of Kratom plant material

Fifty commercial products labeled as kratom were obtained from vendors within the US. A table with details about these products is available in the supporting information (Table S1). The commercial kratom products were obtained as dried powders and were various shades of grey-green, with the exception of product K49, which was obtained as a cut leaf. All plant material was stored dry at room temperature until time of analysis.

A cultivated plant of kratom *(Mitragyna speciosa*) (coded as K55) was generously donated to the project by Shon Lenzo. The plant was grown from the cutting of a 7-year-old tree from a strain labeled as “rifat.” Prior to shipping to the University of North Carolina Greensboro, the cutting was grown indoors in a greenhouse under a 1000 W high pressure sodium (HPS) LED light for 6 months. Upon receipt, the plant was repotted and transferred to an outdoor location near the University of North Carolina Greensboro, where it was grown for several summer months. Leaves were cut from this plant, dried at room temperature and powdered for extraction using a Wiley Mill Standard Model No. 3 (Arthur Thomas Company). A sample of leaves from this plant, harvested at the coordinates 36°4′53.616″ N 79°47′6.18″ W, was pressed and submitted to the University of North Carolina Herbarium, with accession #670043 (sernecportal.org catalog number NCU00433756).

### Isolation of speciofoline

Kratom plant material (K52, 1 kg) was extracted exhaustively with 2 L of CHCl_3_/CH_3_OH (1:1) and 50 mL of KOH (10%) by maceration over 24 h at room temperature. The mixture was filtered, and the solvent was evaporated under reduced pressure. The dried extract was reconstituted in 1 N HCl solution and hexanes (1:1), transferred to a separatory funnel, and shaken vigorously. After removal of the hexanes phase, the pH of the aqueous phase was adjusted to 9.0 with concentrated NH_4_OH solution. The basic phase was exhaustively extracted with CHCl_3_, and the organic phase was evaporated to dryness to yield 1.0 g of the dried extract. Fractionation of the extract was conducted with normal phase flash chromatography using a silica column (24 g) and a gradient solvent system of hexane–acetone-CH_3_OH over 52 min at a flow rate of 35 mL/min. In total, thirteen pooled fractions were obtained. Fraction 4 was subjected to preparative reversed phase HPLC over a Luna PFP(2) (pentafluorophenyl) column (Phenomenex, 250 mm × 21.2 mm, 5 µm) using a gradient of 70:30 to 100:0 CH_3_OH–H_2_O (10 mM of ammonium acetate in both phases) over 20 min at a flow rate of 20 mL/min. This process yielded 25.0 mg of speciofoline (95% purity based on UPLC-UV), the structure of which was solved by comparison of the calculated ([M + H]^+^ C_22_H_29_N_2_O_5_, 401.2076) and measured (401.2067) *m/z* values and by comparison of key ^1^H-NMR signals (Figure S1 and Table S2) and the ECD spectra (Figure S2) with those reported in the literature^25^.

### Extraction for metabolomics profiling

For each extraction of powdered kratom sample, 50 mg of dried kratom plant material was extracted with 5.0 mL of methanol in a 25 mL scintillation vial. Extracts were shaken at 150 rpm for 12 h and the powder was allowed to settle. The extracts were decanted into clean scintillation vials and dried under nitrogen. Extracts were resuspended in methanol containing 0.125 µg/mL of mitragynine-D_3_ (MilliporeSigma, St. Louis, MO, USA) for analysis. All diluted extracts contained mitragynine-D_3_ at a final concentration of 0.125 µg/mL as an internal standard to monitor the consistency of the instrument performance.

### Ultraperformance liquid chromatography–mass spectrometry (UPLC-MS) analysis of Kratom extracts

Untargeted metabolomics analyses were conducted using an Acquity Ultra-Performance Liquid Chromatography system (Waters, Milford, MA, USA) coupled to a Q Exactive Plus Hybrid Quadrupole-Orbitrap mass spectrometer (Thermo Fisher Scientific, Waltham, MA). A Kinetex F5 column (Phenomenex, 50 mm × 1 mm, 2.6 µm) was employed with a flow rate of 0.6 mL/min and a column temperature of 40 °C. The starting conditions were 65% A, optima grade water with 0.1% formic acid and 2 mM ammonium formate, and 35% B, optima grade methanol with 0.1% formic acid and 2 mM ammonium formate. The gradient is as follows: a slight decrease to 55% A at 0.5 min which is held until 3.0 min, followed by a linear decrease to 20% A at 10 min down to 0% A at 11.0 min. The gradient returns to starting conditions at 12 min and that is held for 1 min. A 5 µL injection was employed for all samples using a 5 µL sample loop.

Mass spectrometry analysis utilized a heated electrospray ionization (HESI) source: spray voltage 3.5 kV, capillary temperature 275 °C, sheath gas 55 arbitrary units, auxiliary gas 15 arbitrary units, sweep gas 3 arbitrary units, heater temperature 450 °C, and an RF level of 50. A scan range of 150–900 m*/z* was employed in positive mode with a resolving power of 70,000 and an AGC target of 3E6. MS/MS data were collected on the standards using a normalized collision energy (NCE) of 35 V.

### Metabolomics data processing

The UPLC-MS data were analyzed, aligned, and filtered using MZmine 2.28 software (https://mzmine.github.io/) with a slightly modified version of a previously reported method^42^. The following parameters were used for peak detection of the data: noise level (absolute value), 1 × 10^5^ counts; minimum peak duration 0.5 min; tolerance for *m/z* intensity variation, 20%. Peak list filtering and retention time alignment algorithms were performed to refine peak detection. The *join algorithm* was used to integrate all the chromatograms into a single data matrix using the following parameters: the balance between *m/z* and retention time was set at 10.0 each, *m/z* tolerance was set at 0.001 or 5 ppm, and retention time tolerance was defined as 0.5 min. Once an aligned peak list was created, features (*m/z* and retention time pairs) present in the first blank sample of the run were deleted as a means of blank filtering. The peak areas for individual ions detected in the process replicates and analytical replicates were exported from the data matrix for further analysis. The peak list from MZmine was exported into Excel (Microsoft, Redmond, WA) for further filtering based on relative standard deviation between analytical replicates. Analytical replicates would be expected to have comparable profiles, and similar peak areas for each feature. Ions detected within the analytical replicates with disparate peak areas (based on an RSD cutoff of 25%) were assigned as artefacts of the instrument and excluded from the metabolomics analysis^43^.

Principal component analysis (PCA) was performed using Sirius version 10.0 (Pattern Recognition Systems AS, Bergen, Norway). The 95% confidence intervals were calculated using Hotelling’s T^2^ with the R package ‘car’^44^ using an R script available from https://github.com/joshkellogg/Composite-score.

### Identification and quantitative analysis of alkaloids in kratom plant material

Standard solutions of mitragynine and 7-hydroxymitragynine were purchased from MilliporeSigma (St. Louis, MO, USA) and supplied as methanolic solutions at a concentration of 100 µg/mL. An analytical standard for speciofoline was unavailable for purchase, therefore an aliquot of purified speciofoline isolated as part of this study was dissolved in methanol at a concentration of 100 µg/mL. A 100 µL aliquot of each standard solution was combined with 600 µL of methanol and 100 µL of water to make a stock solution containing 10 µg/mL of each alkaloid of interest. A standard calibration curve was prepared using the stock solution in two-fold dilutions from 5000 ng/mL to 4.9 ng/mL followed by a single 1:10 dilution of the lowest concentration in methanol. All samples were analyzed using the UPLC-MS method previously described. The selected-ion chromatograms for the calculated *m/z* values of the alkaloids of interest (mitragynine, [M + H]^+^  = 399.2284; 7-hydroxymitragynine, [M + H]^+^  = 415.2233; speciofoline, [M + H]^+^  = 401.2076) within a mass error of 5 ppm were plotted, and a calibration curve was constructed as area of these chromatograms versus concentrations. Calibration parameters are provided in Table S3. Identity of the alkaloids in the *M. speciosa* plant material was confirmed by matching retention time and m/z value with that of the standards. The alkaloid concentrations in the kratom extracts were determined using linear regression of these calibration curves. Peak areas were calculated using the Thermo Xcalibur QuanBrowser version 3.0.63 and statistical analysis was performed using RStudio version 1.2.1335.

### Authentication of plant material

Authentication (assignment of genus and species) of kratom products K51, K52 (powdered material), and K55 (vouchered plant specimen) was accomplished using DNA barcoding and maximum likelihood phylogenetic analysis. The most widely recommended loci, the plastid intergenic spacer *trnH-psbA, rbcL*, *matK,* and the nuclear ribosomal internal transcribed spacers (ITS) were employed. These regions have been designated as the official DNA barcodes for the plant kingdom^29,30^.

For DNA extraction from the plant material (K55), a whole leaf sample was ground to a fine powder with a sterile mortar and pestle using liquid nitrogen. Approximately 5 mg of plant powder from the plant (K55) or from commercial sources (K51, K52) was transferred to a bashing bead tube with DNA lysis buffer provided by Zymo Research Quick-DNA plant/seed miniprep DNA extraction kit. The powder in the bashing bead tube was further disrupted and homogenized in a Qiagen TissueLyser LT beadmill for five minutes. For K51 and K52, the samples were separately ground in bashing bead tubes, without treatment with liquid nitrogen. Genomic DNA was then extracted according to kit instructions.

Detailed methods for DNA extraction, PCR amplification, and Sanger sequencing are outlined previously^45^. The partial *rbcL* region was amplified and sequenced using primers rbcLa-F and rbcLa-R^30,46^. Details of PCR primers and thermocycler parameters for all fragments amplified are outlined in Supplementary Table S4.

For identification of kratom plant material via DNA Barcoding, two plastid regions (*rbcL* + *matK*) sequences were utilized in a combined analysis for plant identification by BLAST searching against the BOLDSYSTEMS database.

Uncorrected p-distances were calculated in Geneious, a bioinformatics desktop software package (https://www.biomatters.com) for sequences obtained from the *trnH-psbA* plastid region, which is considered as one of the most variable regions of angiosperm plants^47–50^. Comparisons were made using published sequence data from NCBI GenBank for *Mitragyna* spp., including *M. speciosa* and the newly obtained sequences in this study (Supplementary Table S5). The p-distances were obtained by dividing the number of nucleotide differences by the total number of nucleotides being compared. A cut off proxy of ≥ 98–100% was applied as a criterion to designate similar species; therefore, to be considered the same species based on *trnH-psbA* sequence comparison, the taxa being compared should have ≥ 98% sequence similarity (with 1–2% intraspecific variation or differences).

Maximum likelihood and Bayesian analysis was performed separately for both the combined analysis using *matK* + *trnH-psbA,* and the nrDNA ITS region to place the kratom samples (K51, K52, and K55) into a phylogenetic framework with authentic published sequences of *M. speciosa*^48–52^ using methods outlined previously^53^ (see Supplementary for details)*.*

*Nauclea officinalis* (Rubiaceae) was used as an outgroup taxon in both *matK* + *trnH-psbA* and ITS region analysis, respectively. The sequence data obtained from samples K51, K52, and K55 are deposited in GenBank and accession numbers are provided in Table S6.

### Cytochrome P450 (CYP) inhibition assay

Mitragynine and 7-hydroxymitragynine (purchased), speciofoline (the isolated alkaloid), and three methanolic kratom extracts (prepared from commercial products K50, K51, and K52; Table 1), were tested as inhibitors of CYP2C9, CYP2D6, and CYP3A activities using a cocktail approach^54^. Diclofenac (4 µM), dextromethorphan (4 µM), and midazolam (2 µM) were used as probe substrates for CYP2C9, CYP2D6, and CYP3A activities, respectively. Kratom extract (2, 10, 20 µg/mL) or alkaloid (1, 10, 100 µM) was incubated with human liver microsomes (0.05 mg/mL) in 100 mM potassium phosphate buffer, pH 7.4. Positive control inhibitors included sulfaphenazole (1 µM; CYP2C9), quinidine (2 µM; CYP2D6), and ketoconazole (0.1 µM; CYP3A). Final organic solvent (methanol) concentration in each incubation was limited to 0.8% v/v. After equilibrating the mixtures for 5 min at 37 °C, the reaction was initiated by addition of NADPH (1 mM final concentration) and incubated for 10 min (CYP2C9 and CYP2D6 activities) or 2 min (CYP3A activity) at 37 °C. Reactions were quenched with 2 volumes of cold methanol containing internal standard (alprazolam, 0.1 µM). The quenched mixtures were centrifuged at 2270 × *g* for 10 min, and the supernatants were subjected to UPLC-MS/MS analysis for quantification of 4′-hydroxydiclofenac, dextrorphan, and 1′-hydroxymidazolam. The percent of CYP inhibition in the presence of the test article was calculated relative to solvent control.

The CYP-mediated metabolites (4′-hydroxydiclofenac, dextrorphan, and 1′-hydroxymidazolam) were quantified using a QTRAP system (AB Sciex, Framingham, MA), operating in positive electrospray ionization mode, coupled to a Shimadzu Nexera X2 UHPLC (Shimadzu Corporation, Tokyo, Japan). Chromatographic separation was achieved using an Acquity C18 column (3 µm, 50 × 2.1 mm, Thermo Scientific, Waltham, MA) with a guard column, heated to 40 °C, and a binary gradient at a flow rate of 0.5 mL/min. The mobile phase consisted of 0.1% formic acid in water (A) and 0.1% formic acid in methanol (B). The following gradient was applied: 0–0.4 min, 10% B; 0.4–1.0 min, 10–95% B; 1.0–2.0 min, 95% B; 2.0–2.1 min, 95%-10% B; and 2.1–3.0 min, 10% B. The following multiple reaction monitoring transitions were used: *m/z* 312.0 → 231.0 (4′-hydroxydiclofenac), *m/z* 258.2 → 157.2 (dextrorphan), *m/z* 342.0 → 324.0 (1′-hydroxymidazolam), and *m/z* 309.1 → 281.0 (alprazolam). All analyte concentrations were quantified using MultiQuant software (version 2.1.1; AB Sciex; https://sciex.com/products/software/multiquant-software) by interpolation from calibration curves prepared from relevant matrix-matched standards over a range of concentrations (1.37–1000 nM).

### cAMP accumulation assay

The cAMP Hunter eXpress OPRM1 CHO-K1 GPCR assay (Eurofins DiscoverX Corporation, St. Charles, MO, USA) was performed according to manufacturer’s instructions. Briefly, cells were seeded in 100 µL cell plating reagent in 96 well plates and allowed to incubate at 37 °C (5% CO_2_, 95% relative humidity) for 24 h. Medium was removed from cells, and cells were washed with 30 μL of cell assay buffer. Each compound was assessed using an 11 point fivefold serial dilution with a starting concentration of 10 μM. Aliquots of compound/forskolin solution were added to cells (final concentrations were 20 μM forskolin, and 0.4% DMSO) and incubated at 37 °C and 5% CO_2_ for 30 min. Next, cAMP antibody reagent detection solution was added to each well according to manufacturer’s instructions at room temperature and protected from light for 1 h. Next, enzyme acceptor solution was added and the solution allowed to incubate at room temperature and protected from light for 3 h. Luminescence was quantified using a SpectraMax iD5 multi-mode microplate reader with SoftMax Pro software (version 7.1, Molecular Devices, San Jose, CA, https://www.moleculardevices.com/products/microplate-readers/acquisition-and-analysis-software/softmax-pro-software). Data were normalized to vehicle and forskolin only control values and analyzed using nonlinear regression with Prism 8.4 (Graphpad, San Diego, CA).

### β-arrestin recruitment assay

The PathHunter eXpress OPRM1 CHO-K1 β-Arrestin GPCR assay (Eurofins DiscoverX Corporation) was performed according to the manufacturer’s protocol. Briefly, cells were seeded in 100 µL cell plating reagent in 96 well plates and allowed to incubate at 37 °C (5% CO_2_, 95% relative humidity) for 48 h. Each compound was assessed using an 11 point fivefold serial dilution with a starting concentration of 10 µM. Aliquots of compound solution were added to cells (final concentrations were 20 μM forskolin, and 0.4% DMSO) and incubated at 37 °C and 5% CO_2_ for 30 min. Detection solution was then added to cells and incubated at room temperature protected from light for 1 h. Luminescence was quantified using a SpectraMax iD5 multi-mode microplate reader with SoftMax Pro software (Molecular Devices, San Jose, CA). Data were normalized to control values and analyzed using nonlinear regression to determine maximal and EC_50_ values (Prism 8.4 for Mac; Graphpad San Diego, CA).

### Binding affinity to µ-opioid receptors

HEK293 cells expressing human µ, δ- or κ-opioid receptors were washed in ice cold PBS and scraped from cell culture plates in cold 50 mM Tris–HCl buffer, pH 7.4. Cells were pelleted at 5200 × *g* for 10 min at 4 °C. Supernatant was discarded and the pellet was washed with more Tris–HCl buffer, sonicated, and centrifuged at 24,000 × g for 40 min at 4 °C. The pellet was re-suspended in Tris–HCl buffer, sonicated, and aliquoted for storage at − 80 °C until use. A Pierce BCA Protein Assay was utilized to determine protein concentration in the membrane, according to manufacturer’s instructions. Membranes were evaluated in saturation experiments to determine the optimal concentration for radioligand binding and functional assays. A competitive binding assay was utilized to determine the K_i_ of each compound similar to previous reports^55,56^. For this, 25 μg of cell membrane was diluted in 50 mM Tris–HCl, pH 7.4 and pipetted into a 96 well plate. Test compounds were reconstituted in DMSO and added to the reaction plate.[^3^H]-DAMGO, [^3^H]-U69-593 or [^3^H]-deltorphin (for µ-, ƙ-, and δ-opioid receptors respectively) was then added at a final concentration of 2–4 nM (Perkin Elmer, Waltham, MA, USA). The reaction was incubated for 1 h at room temperature prior to transfer onto GF/B filter plates (Perkin Elmer). The plates were washed 10 × with cold buffer and dried for 15 min at 50 °C. MicroScint20 was then added to each well, the plates were sealed, and the counts per minute were quantified on the TopCount NXT Microplate Scintillation counter (Perkin Elmer). The percent displacement of the radiolabeled compound was calculated as described^57^. Unlabeled DAMGO was used to determine non-specific binding control counts, while vehicle (DMSO) was added to determine total radioligand binding on each plate. A series of three-fold dilutions were used for each test compound starting at 10 μM. A control curve of naloxonewas included on each plate for internal control.

## Supplementary information

Supplementary Information

## Data Availability

Raw mass spectrometric data have been made publicly available through MassIVE (MassIVE ID: MSV000086288, https://doi.org/10.25345/C59V0X). All other data are available by request to the corresponding author.
