# Supplementary material for: Chemical composition and biological effects of kratom (Mitragyna speciosa): In vitro studies with implications for efficacy and drug interactions
Source: Sci Rep. 2020 Nov 5;10:19158. doi: 10.1038/s41598-020-76119-w (PMC7645423; doi:10.1038/s41598-020-76119-w)
Supplement: Supplementary file 1 — Supplementary Information [file 41598_2020_76119_MOESM1_ESM.docx]

Chemical composition and biological effects of kratom *(Mitragyna speciosa*): In vitro studies with implications for efficacy and drug interactions

Todd, D. A.^1^; Kellogg, J. J.^1,2^; Wallace, E. D.^1,3^; Khin, M.^1^, Flores-Bocanegra, L.^1^; Tanna, R. S.^4^; McIntosh, S.^5^; Raja, H. A.^1^; Graf, T. N.^1^; Hemby, S. E.^5^; Paine, M. F.^4^; Oberlies, N. H.^1^; Cech, N. B.^1^*

1. Department of Chemistry and Biochemistry; The University of North Carolina Greensboro; Greensboro, NC 27402
2. Department of Veterinary and Biomedical Sciences, Pennsylvania State University; University Park, PA 16802
3. Department of Chemistry; The University of North Carolina Chapel Hill; Chapel Hill, NC 27599
4. Department of Pharmaceutical Sciences; Washington State University; Spokane, WA 99202
5. Department of Basic Pharmaceutical Sciences; High Point University; High Point, NC 27268

*corresponding author; email: [nadja_cech@uncg.edu](mailto:nadja_cech@uncg.edu); UNC Greensboro Department of Chemistry and Biochemistry; 435 Sullivan Bldg.; 301 McIver St.; Greensboro, NC 27402

**Contents**

**Supplementary Table S1.** Commercial Kratom (*Mitragyna speciosa*) products acquired for study.

**Supplementary Table S2.** ^13^C and ^1^H NMR data for speciofoline.

**Supplementary Figure S1.** ^13^C and ^1^H NMR spectra for speciofoline

**Supplementary Figure S2.** ECD spectra for speciofoline.

**Supplementary Table S3.** Validation Parameters for *Mitragyna speciosa* alkaloid quantitation. Calculations were based on triplicate analyses of 12 concentrations of standards of each alkaloid using LC-MS.

**Supplementary Figure S3.** Comparison of alkaloid profiles obtained with LC-MS analysis.

**Supplementary Figure S4.** Graphical overview of the BLAST results (April 2020) database using *rbcL + matK*.

**Supplementary Figure S5.** Graphical overview of the BLAST results (April 2020) in BOLD database using *rbcL + matK*  (core loci of the Consortium for the Barcode of Life; CBOL).

**Supplementary Figure S6.** Graphical overview of the BLAST results (April 2020) in BOLD database using *rbcL + matK*  (core loci of the Consortium for the Barcode of Life; CBOL).

**Supplementary Table S4**. Primers and PCR protocols for plant identification

**Supplementary Table S5a and S5b.** Uncorrected p-distances from *trnH-psbA* region indicating that kratom samples barcoded in our study have zero nucleotide differences and higher sequence similarity with *Mitragyna speciosa*.

**Supplementary Table S6.** GenBank accession numbers for published taxa used in BLAST search, distance and phylogenetic analyses. Data sequenced in this study are in bold.

**Supplementary Figure S7.** Phylogenetic tree (PhyML; -lnL = 16324.9) inferred from the DNA sequence data from combined analysis of the *matK* and *trnH-psbA* regions (1135 bp).

**Supplementary Figure S8.** Phylogenetic tree (PhyML; -lnL = 1303.65) inferred from the DNA sequence data from the Internal Transcribed Spacer region (nrDNA ITS).

**Supplementary Methods of Maximum Likelihood and Bayesian Analysis**

**Supplementary Table S1.** Commercial Kratom (*Mitragyna speciosa*) products acquired for study.

| **Code** | **Product Name** | **Color^a^** | **Variety^b^** |
| --- | --- | --- | --- |
| **K01** | Red Maeng Da Powder | Red | Maeng Da |
| **K02** | Horned Red Vein | Red | Horned |
| **K03** | Red Sunda | Red | Sundra |
| **K04** | Red Thai | Red | Thai |
| **K05** | White Maeng Da | White | Maeng Da |
| **K06** | Yellow Horn | Yellow | Horn |
| **K07** | Indo Super White | White | Indonesian |
| **K08** | Red Bentuangie | Red | Bentuangie |
| **K09** | Malaysian Super Green | Green | Malay |
| **K10** | Horned Super Red | Red | Horned |
| **K11** | Green Sundanese | Green | Sundanese |
| **K12** | Sumatra Red Vein | Red | Sumatra |
| **K13** | Borneo White Vein |  | Borneo |
| **K14** | Borneo White | White | Borneo |
| **K15** | Maeng Da White | White | Maeng Da |
| **K16** | Yellow Vietnam | Yellow | Vietnam |
| **K17** | Bali |  | Bali |
| **K18** | Red Sumatra | Red | Sumatra |
| **K19** | Green Bali | Green | Bali |
| **K20** | OG Bali Powder |  | Bali |
| **K21** | Red Elephant | Red | Elephant |
| **K22** | Green Malay | Green | Malay |
| **K23** | Red Hulu | Red | Hulu |
| **K24** | Green Indonesian | Green | Indonesian |
| **K25** | Red Maeng Da | Red | Maeng Da |
| **K27** | Yellow Indonesian | Yellow | Indonesian |
| **K28** | White Malay | White | Malay |
| **K29** | Red Horn | Red | Horn |
| **K30** | Green Maeng Da | Green | Maeng Da |
| **K31** | White Jongkong | White | Jongkong |
| **K32** | Red Indonesian | Red | Indonesian |
| **K33** | Yellow Bali | Yellow | Bali |
| **K34** | Maeng Da Red | Red | Maeng Da |
| **K35** | Red Vein Kali | Red | Kali |
| **K36** | Bali Gold | Gold | Bali |
| **K37** | Red Bali | Red | Bali |
| **K38** | Horned White | White | Horned |
| **K39** | Maeng Da |  | Maeng Da |
| **K40** | Red Jongkong | Red | Jongkong |
| **K41** | Maeng Da |  | Maeng Da |
| **K42** | Red Malay | Red | Malay |
| **K43** | Maeng Da |  | Maeng Da |
| **K44** | Green Jongkong | Green | Jongkong |
| **K46** | Thai Red Vein | Red | Thai |
| **K47** | White Maeng Da | White | Maeng Da |
| **K48** | Red Kali | Red | Kali |
| **K49** | Cut leaf | Green | Maeng Da |
| **K50** | Super Horned Red (new batch K10) | Red | Horned |
| **K51** | Yellow Indonesian (new batch K27) | Yellow | Indonesian |
| **K52** | White Jongkong (new batch K31) | White | Jongkong |
| **K55** | Living plant from Shon Lenzo  (accession # 670043)^c^ | 670043 | Rifat |

1. Color is as indicated on product packaging and does not reflect actual color of the plant material received. Samples K01 through K48, K50, K51, and K52 were obtained as powders. All powdered kratom material was grey-green in color and varied slightly in shade. Sample K49 was dried cut leaf material. Sample K55 was a dried leaf from a living plant obtained from Shon Lenzo.
2. Variety is included on the packaging
3. 670043 is the herbarium accession number for sample K55 to the University of North Carolina Herbarium, the digital voucher can be accessed through the SERNAC portal ([sernecportal.org](http://sernecportal.org/) catalog number NCU00433756).

**Supplementary Table S2.** ^13^C and ^1^H NMR data for speciofoline (100 and 400 MHz, respectively, CDCl_3_)

| **Position** | **δ_C_, type ^a^** | **δ_H_ (*J* in Hz)^a^** | **δ_H_ (*J* in Hz)^b^** |
| --- | --- | --- | --- |
| 2 | 180.2, C | - |  |
| 3 | 63.8, CH | 3.06, dd (11.7, 3.6) |  |
| 5 | 53.1, CH_2_ | 3.43, td (9.2, 3.2)  2.67, m |  |
| 6 | 33.9, CH_2_ | 2.40, ddd (13.8, 10.9, 3.3)  2.14, m |  |
| 7 | 57.3, C | - |  |
| 8 | 116.7, C | - |  |
| 9 | 154.6, C | - |  |
| 10 | 111.9, CH | 6.35, d (7.6) | 6.32, d |
| 11 | 129.6, CH | 7.05, t (8.0) | 7.03, t |
| 12 | 101.1, CH | 6.53, d (8.3) | 6.51, d |
| 13 | 140.7, C | - |  |
| 14 | 31.6, CH_2_ | 1.49, ddd (13.7, 12.1, 6.5)  1.40, m |  |
| 15 | 31.1, CH | 3.27, t (5.7) |  |
| 16 | 111.0, C | - |  |
| 17 | 159.8, CH | 7.38, s | 7.36, s |
| 18 | 12.3, CH_3_ | 0.83, t (7.4) | 0.82, t |
| 19 | 24.2, CH_2_ | 1.21, qd (7.3, 2.5) |  |
| 20 | 38.9, CH | 1.90, dt (12.3, 5.3) |  |
| 21 | 53.6, CH_2_ | 3.06, dd (11.7, 3.6)  2.89, t (11.6) |  |
| 22 | 169.6, C | - |  |
| 17-OCH_3_ | 61.7, CH_3_ | 3.79, s | 3.77, s |
| 22-OCH_3_ | 51.7, CH_3_ | 3.65, s | 3.58, s |
| NH | - | 8.44, s |  |

**^a^**Experimental NMR data for isolated speciofoline

**^b^**Data reported for speciofoline (Ref: *Phytochemistry* 1975, vol. 14, pp. 557-563)

**Supplementary Figure S1.** ^13^C and ^1^H NMR spectra for speciofoline (CDCl_3_, 400 and 100 MHz, respectively)


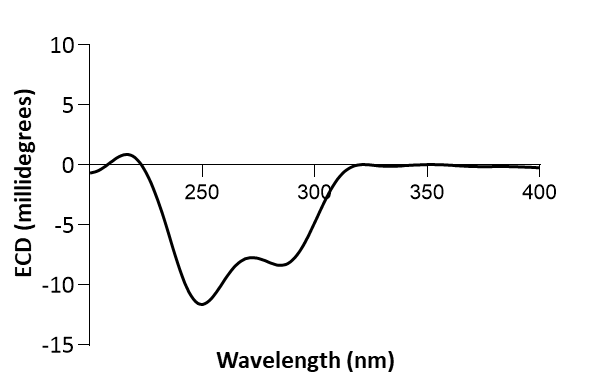


**Supplementary Figure S2.** ECD spectra for speciofoline, matching with the previously reported (Ref: *Phytochemistry* 1975, vol. 14, pp. 557-563)

**Supplementary Table S3.** Validation Parameters for *Mitragyna speciosa* alkaloid quantitation. Calculations were based on triplicate analyses of 12 concentrations of standards of each alkaloid using LC-MS.

| **Validation parameter** | **Mitragynine** | **7-Hydroxymitragynine** | | **Speciofoline** |
| --- | --- | --- | --- | --- |
| **Correlation coefficient** | 0.999 | | 0.999 | 1.000 |
| **Standard error** | 4.86 × 10^7^ | | 1.51 x 10^5^ | 5.60 x 10^6^ |
| ***y*-Intercept** | (2.5 ± 1.3) × 10^7^ | | (8.1 ± 5.7) × 10^4^ | (-3.0 ± 13.2) × 10^5^ |
| **Slope of regression line** | (1.482 ± 0.012) × 10^9^ | | (4.133 ± 0.028) × 10^8^ | (1.289 ± 0.005) × 10^9^ |
| **Limit of detection**  **(LOD)^a^** | 0.49 ng/mL | | N.D. | 0.59 ng/mL |
| **Lower limit of quantitation**  **(LLOQ)^b^** | 78 ng/mL | | 0.49 ng/mL | 4.9 ng/mL |
| **Upper limit of quantitation (ULOQ)^c^** | 2500 ng/mL | | 39.1 ng/mL | 625 ng/mL |

^a^ LOD was defined as the concentration that provided a signal to noise ratio of 3:1 with noise determined by injection of a solvent blank.

^b^LLOQ was defined as the lowest concentration of standard in the calibration curve that provided a residual of less than 20%.

^c^ULOQ was defined as the highest concentration of standard in the calibration curve that provided a residual of less than 20%.


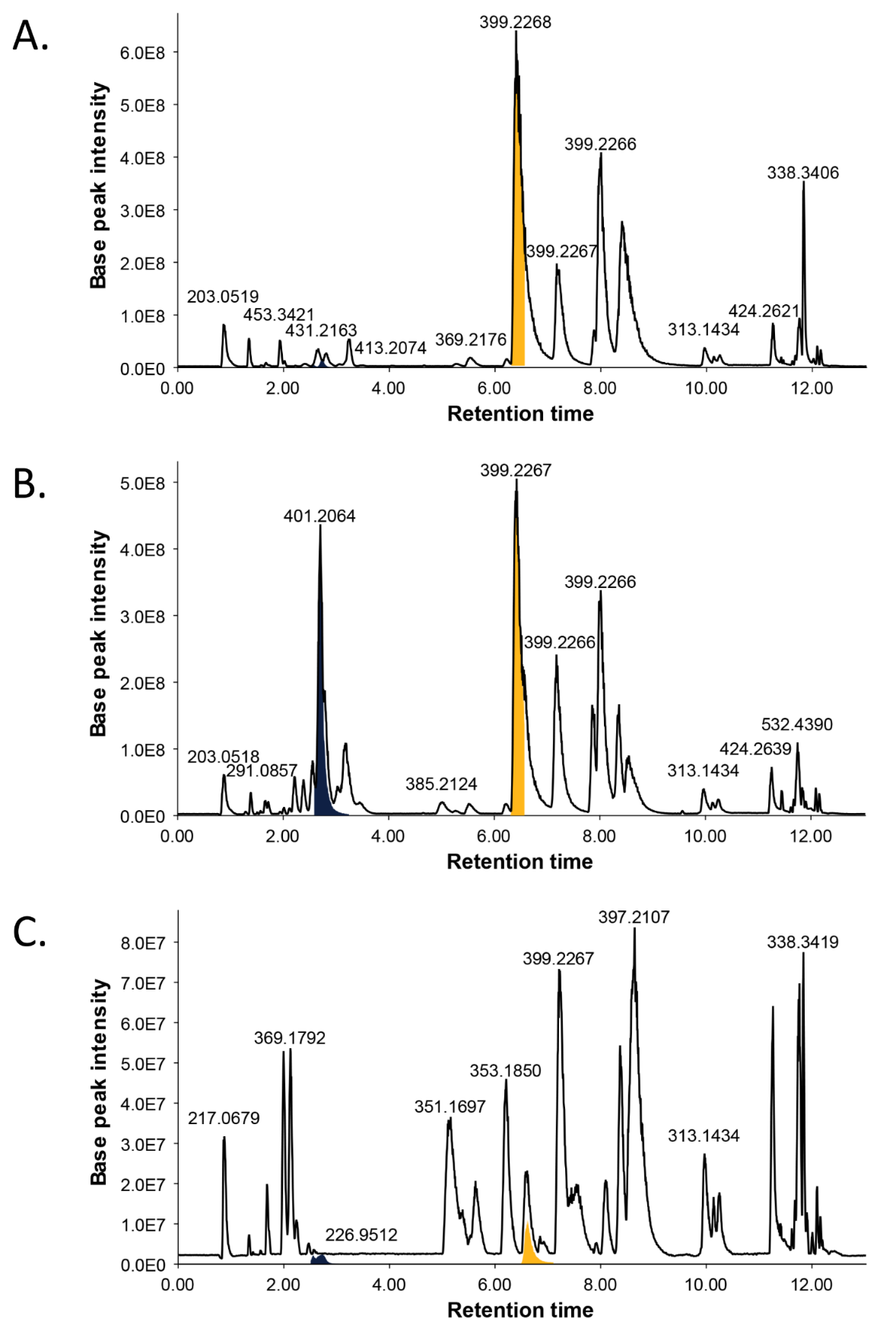


**Supplementary Figure S3.** Comparison of alkaloid profiles obtained with LC-MS analysis for (A) K51, a commercial kratom *(Mitragyna speciosa*) sample from the low speciofoline group, (B) K52 a commercial kratom *(M. speciosa*) sample from the high speciofoline group, and (C) K55 the cultivated *M. speciosa* sample obtained from a supplier in Ohio. Numbers above peaks indicate the *m/z* value for the most abundant ion detected at that retention time. The shaded area highlighted in yellow represents the peak area for the selected ion trace of the [M+H]^+^ ion for the alkaloid mitragynine (*m/z* 399.2259), while the peaks highlighted in blue represent the peak area for the selected ion trace of the [M+H]^+^ ion for the alkaloid speciofoline (401.2057). Note that all chromatograms are normalized to the most abundant peak; the abundance of mitragynine in K55 (panel C) is far lower than that in K51 (panel A) and K52 (panel B).


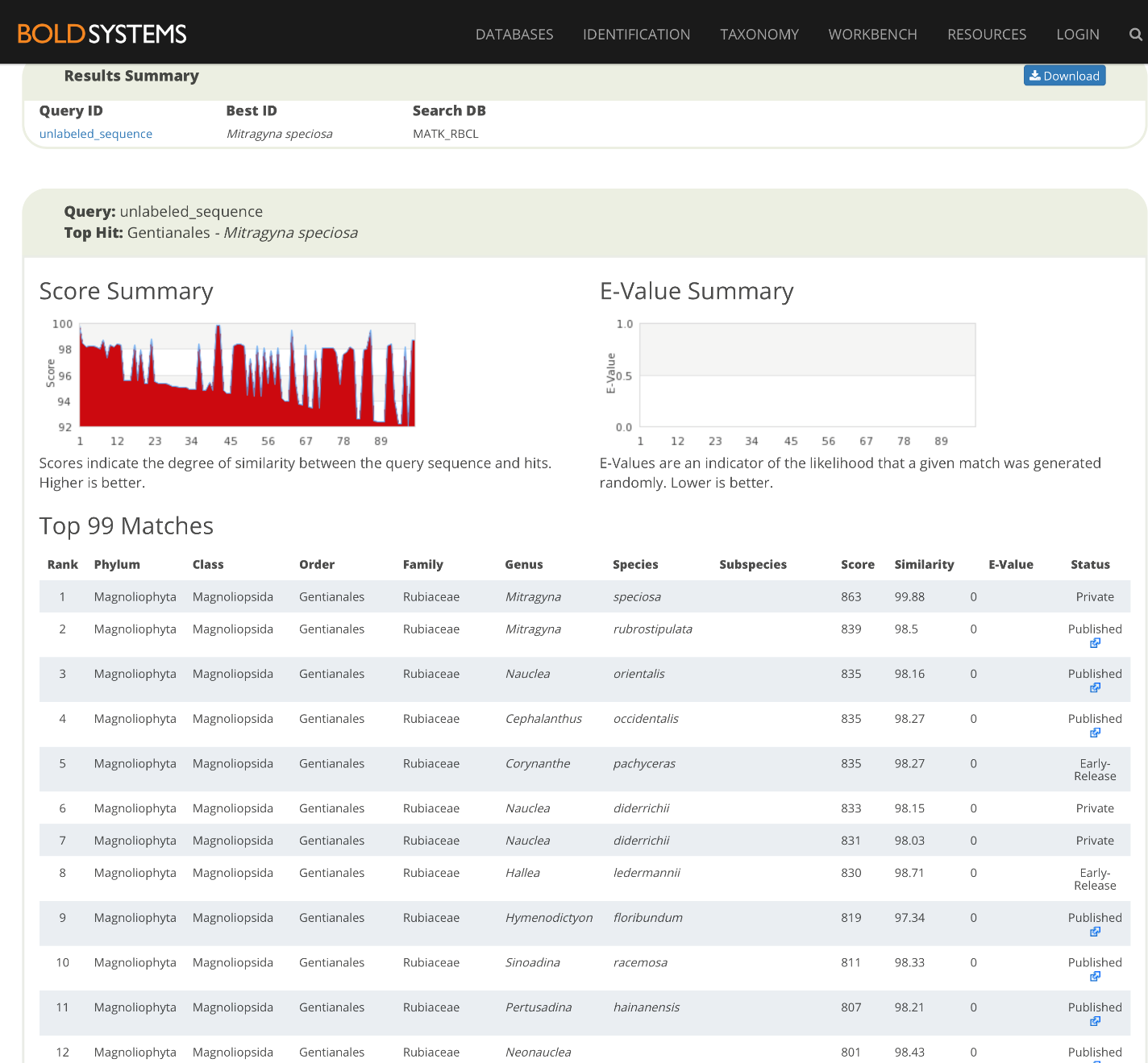


**Supplementary Figure S4.** Graphical overview of the BLAST results (April 2020) in BOLD database using *rbcL + matK*  (core loci of the Consortium for the Barcode of Life; CBOL). Sample K51 shows ≥ 99% similarity with *Mitragyna speciosa.* Only the top 10 results are shown.


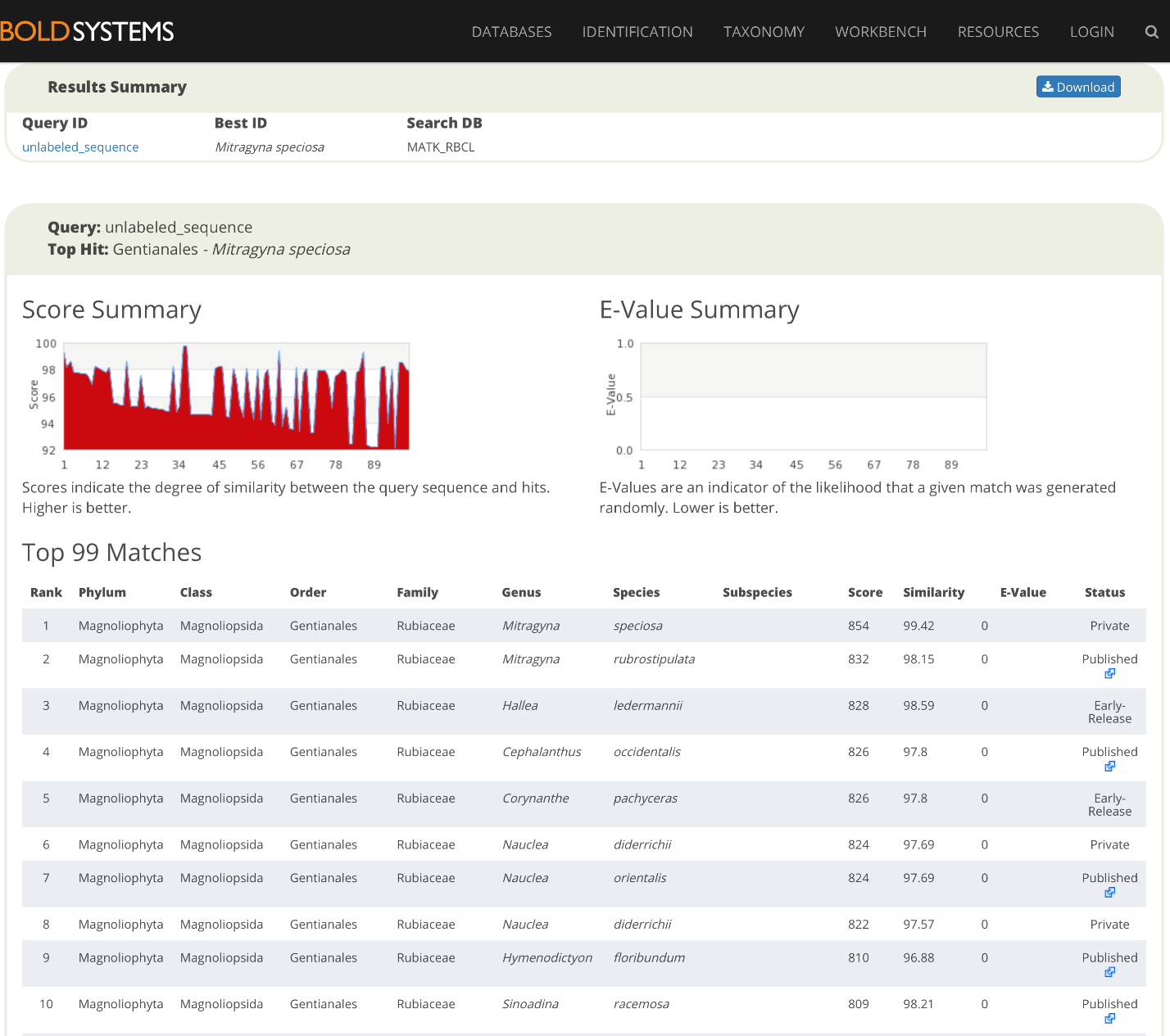


**Supplementary Figure S5.** Graphical overview of the BLAST results (April 2020) in BOLD database using *rbcL + matK*  (core loci of the Consortium for the Barcode of Life; CBOL). Sample K52 shows ≥ 99% similarity with *Mitragyna speciosa.* Only the top 10 results are shown.


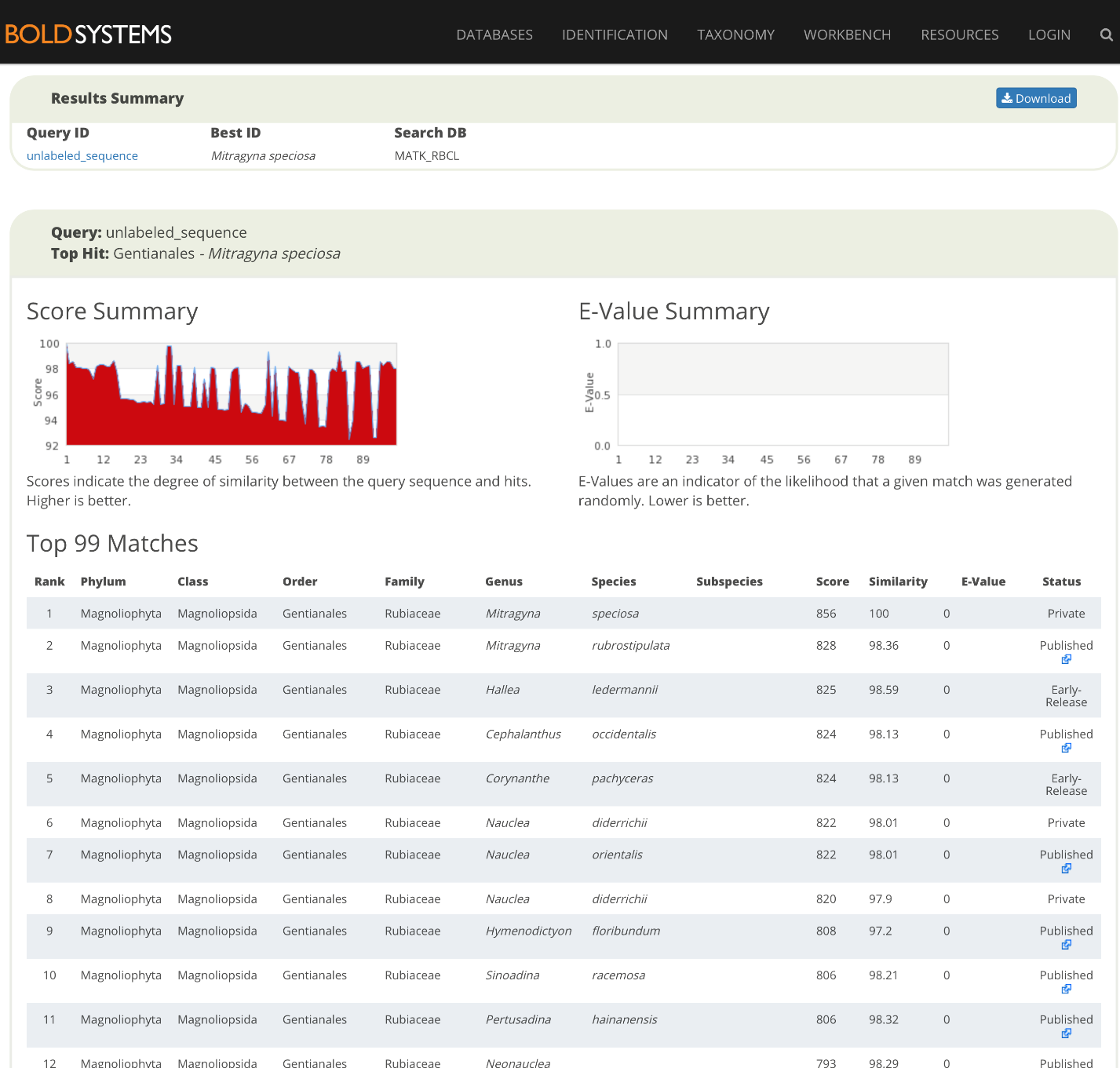


**Supplementary Figure S6.** Graphical overview of the BLAST results (April 2020) in BOLD database using *rbcL + matK*  (core loci of the Consortium for the Barcode of Life; CBOL). Sample K55 shows 100% similarity with *Mitragyna speciosa.* Only the top 10 results are shown.

**Supplementary Table S4**. Primers and PCR protocols for plant identification (Modified from ^1^)

| **Locus** | **Primer** | **Primer Sequence 5'-3'** | **Direction** |  | **PCR protocol*** |
| --- | --- | --- | --- | --- | --- |
| ribulose-bisphosphate carboxylase gene (*rbcL*) | rbcLa-F  rbcLa-R | ATGTCACCACAAACAGAGACTAAAGC  GTAAAATCAAGTCCACCRCG | Forward  Reverse |  | 1. 98˚C – 45 sec  2. 98˚C – 10 sec  3. 55˚C – 30 sec  4. 72˚C – 40 sec  5. Repeat 2–4 for 35 cycles  6. 72˚C – 10 min  7. 4˚C on hold |
| The chloroplast maturase K gene  (*matK*) | matK-xf  matK-MALP | TAATTTACGATCAATTCATTC  ACAAGAAAGTCGAAGTAT | Forward  Reverse |  | 1. 98˚C – 45 sec  2. 98˚C – 10 sec  3. 54˚C – 30 sec  4. 72˚C – 40 sec  5. Repeat 2–4 for 35 cycles  6. 72˚C – 10 min  7. 4˚C on hold |
| The chloroplast intergenic region (*trnH-psbA*) | psbA  trnH | GTTATGCATGAACGTAATGCTC  CGCGCATGGTGGATTCACAATCC | Forward  Reverse |  | 1. 94˚C – 5 min  2. 94˚C – 1 min  3. 50˚C – 1 min  4. 72˚C – 2 min  5. Repeat 2–4 for 35 cycles  6. 72˚C – 7 min  7. 4˚C on hold |
| The internal transcribed spacer (ITS) of nuclear ribosomal DNA | ITS-u1  ITS-u4 | GGAAGKARAAGTCGTAACAAGG  RGTTTCTTTTCCTCCGCTTA | Forward  Reverse |  | 1. 94˚C – 4 min  2. 94˚C – 30 sec  3. 55˚C or 58˚C – 40 sec  4. 72˚C – 1 min  5. Repeat 2–4 for 34 cycles  6. 72˚C – 10 min  7. 4˚C on hold |

**Supplementary Table S5a and S5b.** Uncorrected p-distances from *trnH-psbA* region indicating that kratom samples barcoded in our study have zero nucleotide differences and higher sequence similarity with *Mitragyna speciosa*. Regions with N at the beginning and end of the nucleotide alignment were not taken into consideration for uncorrected p-distances. Comparisons were made using the listed species. *Mitragyna speciosa,* Thailand, LC334417; *Mitragyna speciosa,* voucher A2706*,* MH069946; *Mitragyna rotundifolia,* Thailand, LC334419; *Mitragyna parvifolia* India, MPF1, JX856911; *Mitragyna parvifolia,* India, 400, JX856910; *Mitragyna hirsuta,* Thailand, LC334420; and *Mitragyna diversifolia,* Thailand, LC334418.

**(a) Percent Sequence similarity**

**
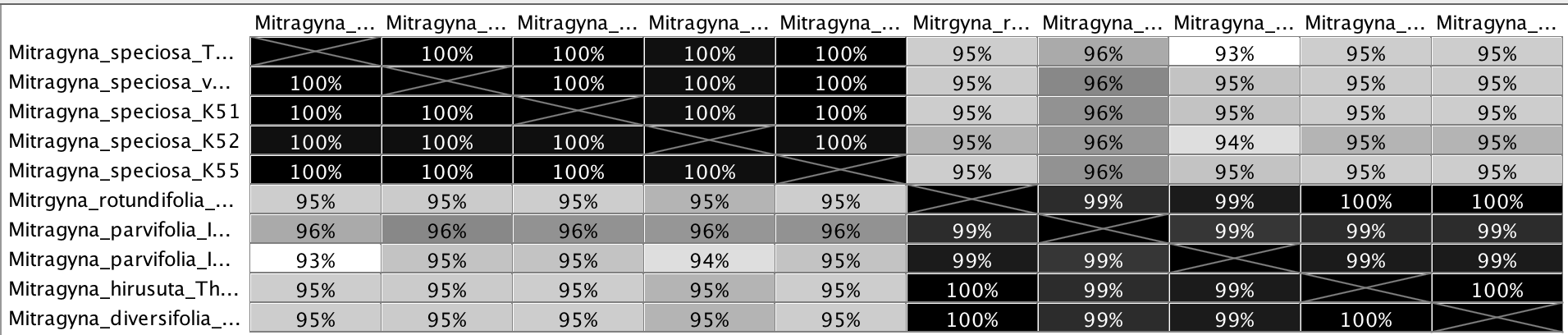
**

**(b) Number of differing nucleotides**

**
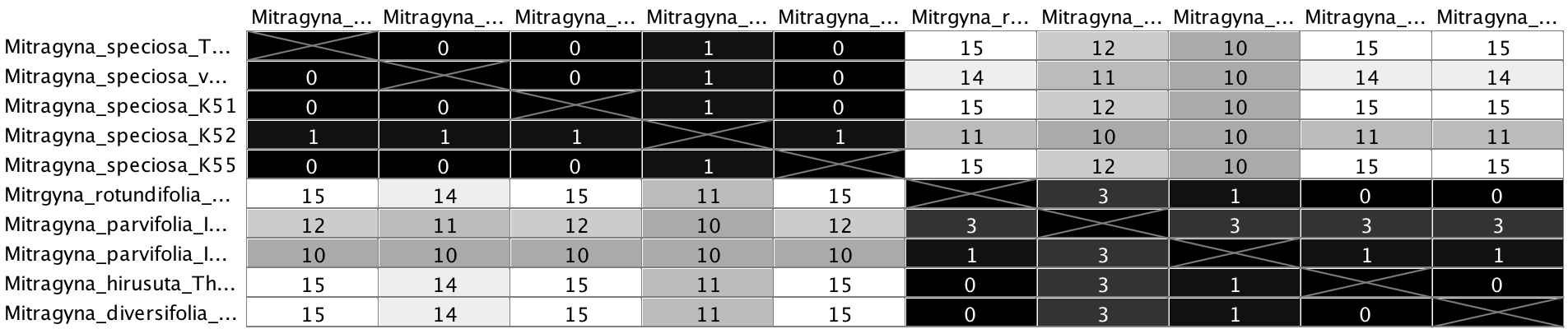
**

**Supplementary Table S6.** GenBank accession numbers for published taxa used in BLAST search, distance and phylogenetic analyses. Data sequenced in this study are in bold.

| **Taxa** | **Specimens** | **ITS** | ***matK/rbcL*** | ***trnH-psbA*** |
| --- | --- | --- | --- | --- |
| *Mitragyna speciosa* | Thailand | AB249645 | LC334409 | LC334417 |
| *Mitragyna speciosa* | voucher A2706 | - | - | MH069946 |
| *Mitragyna speciosa* | voucher Loefstrand | KC737618 | - | - |
| *Mitragyna speciosa* | BioProject: PRJNA325670 | - | KY085908 | - |
| *Mitragyna speciosa* | K51 | **MT586314** | **MT594137/ MT594134** | **MT594139** |
| *Mitragyna speciosa* | K52 | MT111842 | MT114408 **/ MT594135** | MT114411 |
| *Mitragyna speciosa* | K55 | **MT586315** | **MT594138/ MT594136** | **MT594140** |
| *Mitragyna tubulosa* | voucher Fagerlind & Klackenberg 237 (S) | KC737619 | - | - |
| *Mitragyna rotundifolia* | Thailand1 | AB249648 | LC334411 | LC334419 |
| *Mitragyna rotundifolia* | Thailand2 | AJ346874 | - | - |
| *Mitragyna parvifolia* | India, MPF3 | JX856480 | - | - |
| *Mitragyna parvifolia* | India, MPF2 | JX856479 | - | - |
| *Mitragyna parvifolia* | India, MPF1 | JX856478 | - | JX856911 |
| *Mitragyna parvifolia* | India, 400 | JX856477 | - | JX856910 |
| *Mitragyna parvifolia* | voucher Fagerlind & Klackenberg 400 (S) | KC737617 | - | - |
| *Mitragyna inermis* | specimen voucher Lorence NTBG-95066 | AJ346873 | - | - |
| *Mitragyna hirsuta* | Thailand | AB249647 | LC334412 | LC334420 |
| *Mitragyna diversifolia* | Thailand1 | - | LC334410 | LC334418 |
| *Mitragyna diversifolia* | Thailand2 | AB249646 | - | - |
| **Taxa** | **Specimens** | **ITS** | ***matK/rbcL*** | ***trnH-psbA*** |
| *Mitragyna diversifolia* | Thailand3 | AJ346872 | - | - |
| *Nauclea officinalis* | voucher1087 | MG730972 | - | - |
| *Nauclea officinalis* | voucher YLL Y089 | - | KX526781 | - |


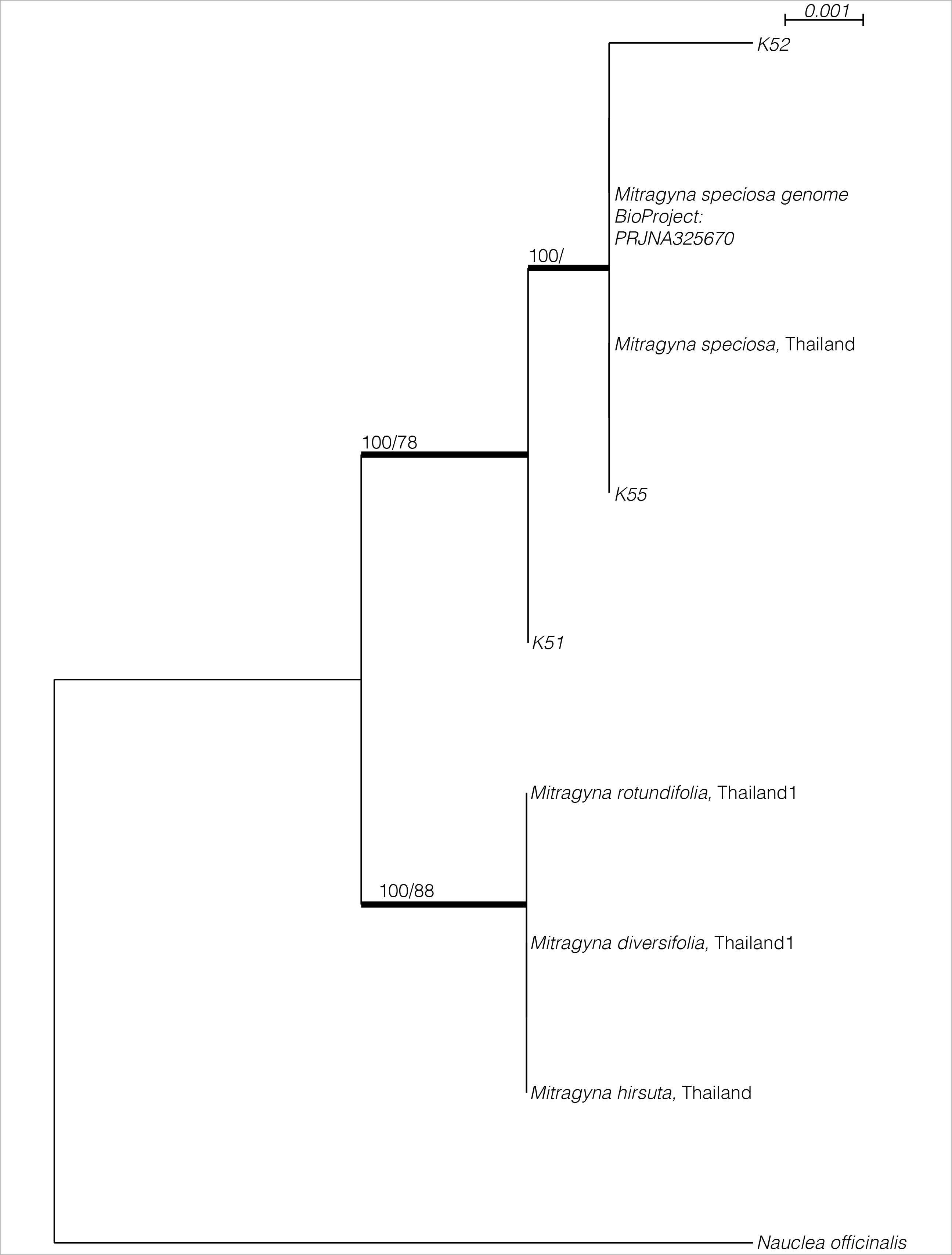


**Supplementary Figure S7.** Phylogenetic tree (PhyML; -lnL = 16324.9) inferred from the DNA sequence data from combined analysis of the *matK* and *trnH-psbA* regions (1135 bp). K51, K52, and K55 form a strongly supported clade with authentic sequence data of *Mitragyna speciosa* with significant bootstrap support and Bayesian posterior probability. The thickened branches indicate significant Bayesian posterior probabilities ≥95% and the numbers refer to RAxML followed by PhyML bootstrap support values ≥70% based on 1000 replicates. Bar indicates nucleotide substitutions per site. The tree was rooted to *Nauclea officinalis.*


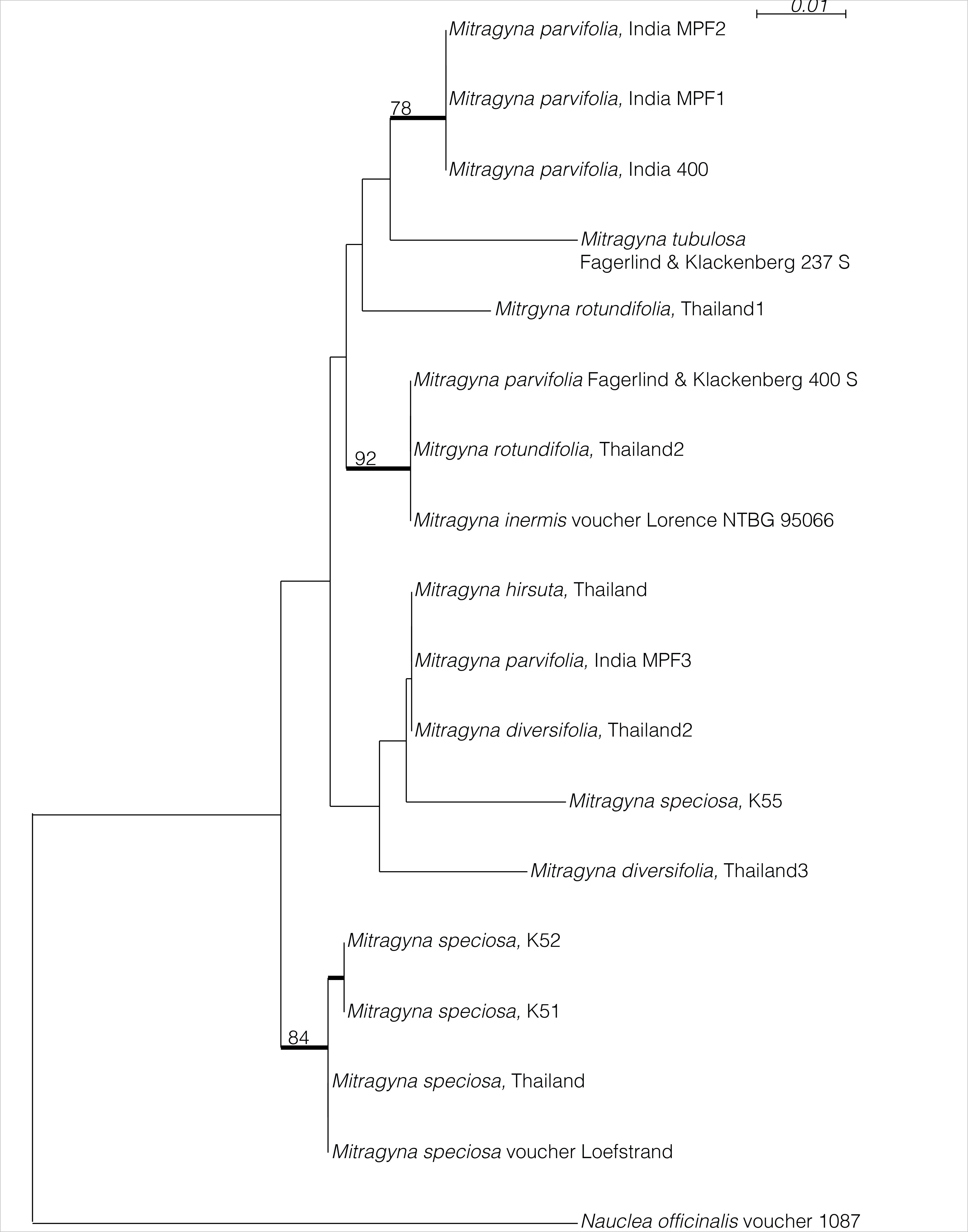


**Supplementary Figure S8.** Phylogenetic tree (PhyML; -lnL = 1303.65) inferred from the DNA sequence data from the Internal Transcribed Spacer region (nrDNA ITS). K51, K52 form a strongly supported clade with authentic sequence data of *Mitragyna speciosa*, however, K55 groups with *Mitragyna diversifolia, M. hirsuta,* and *M. parvifolia,* but with no significant bootstrap support. The thickened branches indicate significant Bayesian posterior probabilities ≥95% and the numbers refer to PhyML bootstrap support values ≥70% based on 1000 replicates. Bar indicates nucleotide substitutions per site. The tree was rooted to *Nauclea officinalis.*

**Supplementary Methods of Maximum Likelihood and Bayesian Analysis**

For the separate and combined datasets, the best-fit model of evolution was tested using JModeltest v. 2.1^2^. For ITS data, Gblocks^3,4^ was used to exclude introns and ambiguously aligned regions. Based on AIC criterion, TPM2uf was selected as the most likely model for the ITS region, while TVM and HKY models were selected for combined *matK +* *trnH-psbA* analysis using PartitionFinder v. 2^5^ on the Portal v. 2.0^6^. Likelihood analyses were run using PhyML^7^ in the program Seaview v. 4.5.3^8^. Bootstrap replicates were performed from a BioNJ starting tree employing the best of nearest neighbor interchange (NNI) and subtree pruning and regrafting (SPR) branch swapping. For combined *matK + trnH-psbA* region Maximum likelihood analyses were also performed using RAxML v. 7.0.4^9^ run on the CIPRES Portal v. 2.0^6^ with the default rapid hill-climbing algorithm and GTR model employing 1000 fast bootstrap searches. Clades with bootstrap values ≥ 70% are considered significant and strongly supported. Bayesian analyses employing a Markov Chain Monte Carlo (MCMC) algorithm was run with MrBayes v. 3.2.4^10^ on the CIPRES Portal v. 2.0 as an additional means of assessing branch support. Four independent chains of MCMC were run for 100 million generations to ensure that the same tree space was being sampled during each analysis and that the trees were not trapped in local optima. Trees were sampled every 1000th generation resulting in 100,000 total trees. Bayesian posterior probabilities (BPP) were determined from a 95% consensus tree generated from the remaining 90,000 trees in time-expiring test versions of PAUP*^11^ after the first 10,000 trees, which extended beyond the burn-in phase in each analysis, were discarded. Clades with posterior probability ≥ 95% were considered significant and strongly supported.

**Suplementary** **References**

1 Flores-Bocanegra, L. *et al.* The chemistry of kratom [*Mitragyna speciosa*]: Updated characterization data and methods to elucidate indole and oxindole alkaloids. *J. Nat. Prod.* **83**, 2165-2177, doi:10.1021/acs.jnatprod.0c00257 (2020).

2 Posada, D. Jmodeltest: Phylogenetic model averaging. *Mol Biol Evol* **25**, 1253-1256, doi:Doi 10.1093/Molbev/Msn083 (2008).

3 Castresana, J. Selection of conserved blocks from multiple alignments for their use in phylogenetic analysis. *Mol Biol Evol* **17**, 540-552 (2000).

4 Talavera, G. & Castresana, J. Improvement of phylogenies after removing divergent and ambiguously aligned blocks from protein sequence alignments. *Syst Biol* **56**, 564-577, doi:Doi 10.1080/10635150701472164 (2007).

5 Lanfear, R., Calcott, B., Ho, S. Y. & Guindon, S. Partitionfinder: Combined selection of partitioning schemes and substitution models for phylogenetic analyses. *Mol Biol Evol* **29**, 1695-1701 (2012).

6 Miller, M. A., Pfeiffer, W. & Schwartz, T. in *In Proceedings of the Gateway Computing Environments Workshop (GCE)* 1–8 (2010).

7 Guindon, S., Delsuc, F., Dufayard, J. F. & Gascuel, O. Estimating maximum likelihood phylogenies with phyml. *Methods Mol Biol* **537**, 113-137, doi:doi: 10.1007/978-1-59745-251-9_6 (2009).

8 Gouy, M., Guindon, S. & Gascuel, O. Seaview version 4: A multiplatform graphical user interface for sequence alignment and phylogenetic tree building. *Mol Biol Evol* **27**, 221-224, doi:doi: 10.1093/Molbev/Msp259 (2010).

9 Stamatakis, A. Raxml-vi-hpc: Maximum likelihood-based phylogenetic analyses with thousands of taxa and mixed models. *Bioinformatics* **22**, 2688-2690, doi:Doi 10.1093/Bioinformatics/Btl446 (2006).

10 Huelsenbeck, J. P. & Ronquist, F. Mrbayes: Bayesian inference of phylogenetic trees. *Bioinformatics* **17**, 754-755 (2001).

11 Paup*: Phylogenetic analysis using parsimony (* and other methods). Version 4 (Sinauer Associates, , Sunderland, Massachusetts 2002).
